# Glucose‐based microbial production of the hormone melatonin in yeast *Saccharomyces cerevisiae*

**DOI:** 10.1002/biot.201500143

**Published:** 2016-01-25

**Authors:** Susanne M. Germann, Simo A. Baallal Jacobsen, Konstantin Schneider, Scott J. Harrison, Niels B. Jensen, Xiao Chen, Steen G. Stahlhut, Irina Borodina, Hao Luo, Jiangfeng Zhu, Jérôme Maury, Jochen Forster

**Affiliations:** ^1^ The Novo Nordisk Foundation Center for Biosustainability, Technical University of DenmarkHørsholmDenmark

**Keywords:** EasyClone vectors, Melatonin, Microbial production, *Saccharomyces cerevisiae*, Serotonin

## Abstract

Melatonin is a natural mammalian hormone that plays an important role in regulating the circadian cycle in humans. It is a clinically effective drug exhibiting positive effects as a sleep aid and a powerful antioxidant used as a dietary supplement. Commercial melatonin production is predominantly performed by complex chemical synthesis. In this study, we demonstrate microbial production of melatonin and related compounds, such as serotonin and *N*‐acetylserotonin. We generated Saccharomyces cerevisiae strains that comprise heterologous genes encoding one or more variants of an *L*‐tryptophan hydroxylase, a 5‐hydroxy‐*L*‐tryptophan decarboxylase, a serotonin acetyltransferase, an acetylserotonin *O*‐methyltransferase, and means for providing the cofactor tetrahydrobiopterin via heterologous biosynthesis and recycling pathways. We thereby achieved de novo melatonin biosynthesis from glucose. We furthermore accomplished increased product titers by altering expression levels of selected pathway enzymes and boosting co‐factor supply. The final yeast strain produced melatonin at a titer of 14.50 ± 0.57 mg L^−1^ in a 76h fermentation using simulated fed‐batch medium with glucose as sole carbon source. Our study lays the basis for further developing a yeast cell factory for biological production of melatonin.

See accompanying commentary by Tao Sun, Lei Chen and Weiwen Zhang DOI10.1002/biot.201500604

**Abbreviations**
AANAT: arylalkylamine‐N‐acetyltransferase (serotonin acetyltransferase)
acetyl‐CoA: acetyl coenzyme A
ACS: acetyl‐CoA synthase
ALD6: aldehyde dehydrogenase
ARO9: aromatic aminotransferase II
ASMT: acetylserotonin *O*‐methyltransferase
BH4/THB: tetrahydrobiopterin
DDC: dopa decarboxylase (5‐hydroxy‐L‐tryptophan decarboxylase)
DHPR: dihydropteridine reductase
ERC1: ethionine resistance conferring gene
FIT: synthetic fed‐batch medium
GCH1: GTP cyclohydrolase I (*FOL2* in *S. cerevisiae*)
5‐HTP: 5‐hydroxytryptophan
MS: methionine synthase
PCBD: pterin‐4‐alpha‐carbinolamine dehydratase (4a‐hydroxytetrahydrobiopterin dehydratase)
PTS: 6‐pyruvoyl‐tetrahydropterin synthase
SAH: S‐adenosyl‐L‐homocysteine
SAH1: S‐adenosyl‐homocysteine hydrosylase
SAM: S‐adenosyl‐L‐methionine
SAM2: S‐adenosyl‐methionine synthetase
SAMS: S‐adenosyl‐methionine synthetase
SPR: sepiapterin reductase
TPH: L‐tryptophan hydroxylase

## 1 Introduction

Melatonin is a natural hormone produced by the pineal gland in the brain 1, 2. In humans, it regulates the body's circadian sleep‐wake cycle 3 and is used as sleep‐aid 4, 5, anti‐oxidant 6, and over‐the‐counter dietary supplements based on melatonin have been available for many years in the US. Moreover studies suggest that the melatonin hormone is also an effective cancer inhibitor 7, 8, can be beneficial in treating neurodegenerative diseases 9, and is useful to fight depression and anxiety 10. The first commercially available melatonin was produced via extraction of pineal glands of animals 1. However, one of the biggest drawbacks of the extraction process is the danger of viral contamination of the animal tissue. Therefore, melatonin is typically chemically synthesized, requiring toxic solvents and catalysts 11, 12, albeit that all of these solvents and chemical intermediates are abundantly available.

This has created a compelling case for a biological production process, which could potentially be the dominant production process for melatonin in the future. In mammals, melatonin is primarily synthesized in the pineal gland in a four‐step pathway using L‐tryptophan as a precursor, which is a downstream metabolite of the intrinsic shikimate pathway 13, 14. For recombinant melatonin production, we chose budding yeast *Saccharomyces cerevisiae* as the host, since the product is intended for food supplement and medical applications. To the best of our knowledge, this is the first report on heterologous production of melatonin from glucose.

## 2 Materials and methods

### 2.1 Strains and chemicals

*Escherichia coli* DH5a was used for cloning, *S. cerevisiae* CEN.PK strains were obtained from Peter Kötter (Johan Wolfgang Goethe‐University Frankfurt, Germany). All oligos are listed in Supporting information, Table S1, DNA BioBricks are listed in Supporting information, Table S2, plasmids are listed in Supporting information, Table S3, and yeast strains are listed in Supporting information, Table S4. EasyClone plasmids used in this study are described in Jensen et al. 15. All chemicals were from Sigma‐Aldrich, except nourseothricin (Werner BioAgents).

### 2.2 Media

*E. coli* was cultivated at 37°C in LB medium. *S. cerevisiae* was cultivated at 30°C on synthetic complete medium or drop‐out media prepared with pre‐mixed drop‐out powders from Sigma‐Aldrich. Mineral medium was prepared as described previously 15. Synthetic fed‐batch (FIT) medium M‐Sc.syn‐1000 was prepared according to manufacturer's instructions (M2P labs GmbH). FIT medium was supplemented with the supplied vitamins solution (final concentration 1% v/v) and the enzyme mix (final concentration 0.5% v/v) immediately prior to use.

### 2.3 Cultivation and analysis

For cultivating yeast strains, single colonies originating from independent transformants were inoculated from a pre‐culture grown in selective minimal medium into 500 µL FIT medium or mineral medium supplemented with 20 g L^−1^ glucose in a 96‐deep well microtiter plate with air‐penetrable lid (EnzyScreen, NL). The microtiter plates were incubated at 30°C with 300 rpm agitation at 5 cm orbit cast, cells were allowed to grow for 72 h. For some experiments, the mineral medium was supplemented with 500 mg L^−1^ methionine or 500 mg L^−1 ^tryptophan. For harvesting, an equal amount of 96% ethanol was added to the supernatant or the total cell suspension, mixed thoroughly, the ethanol‐extracted culture filtered (0.22 µm), and subjected to LC‐ESI‐MS analysis (details of the LC‐ESI‐MS method are found in the Supporting information).

### 2.4 Statistics

For statistical evaluations of the changes in production levels, data were analyzed for significant differences by unpaired two‐tailed *t*‐tests using GraphPad Prism version 6.00 for Windows (GraphPad Software, La Jolla, California, USA).

## 3 Results and discussion

### 3.1 Pathway engineering for sugar‐based recombinant biosynthesis of melatonin in yeast

**Figure 1:**
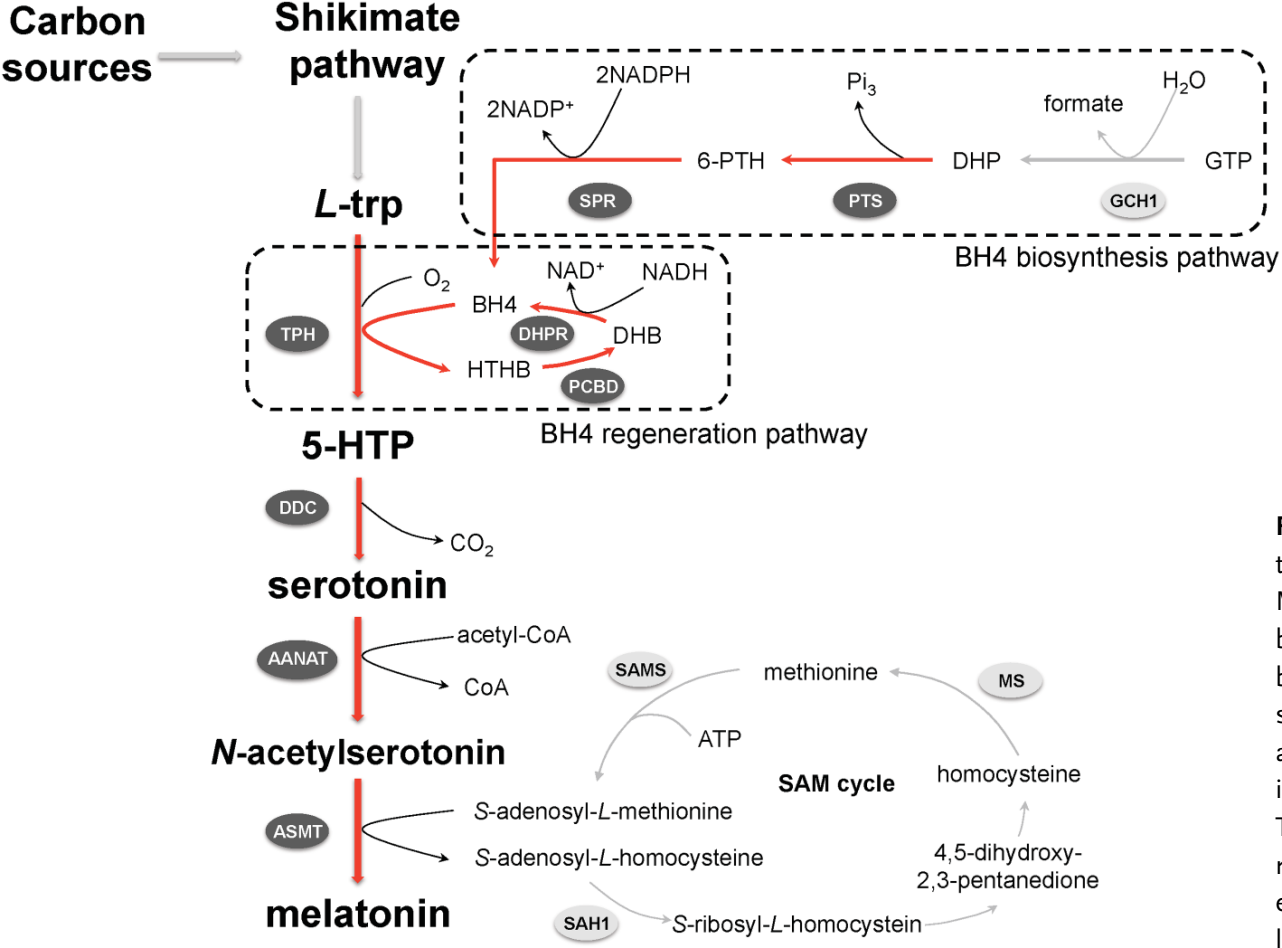
Overview of heterologous melatonin biosynthesis in *Saccharomyces cerevisiae*. Melatonin is synthesized from L‐tryptophan by four enzymatic steps and with the BH4 biosynthesis and regeneration pathways to supply the BH4 cofactor. Enzymatic reactions are indicated by arrows, native reactions are in grey and recombinant ones are in red. The enzymes overexpressed in this study are marked in dark grey with white letters, native enzymes are marked in light grey with black letters.

In this study, a de novo pathway for melatonin production was established in *S. cerevisiae* by the extension of the shikimate synthesis pathway. The biosynthesis pathway is described in Fig. 1, the connections to the intrinsic yeast metabolism are depicted in Supporting information, Fig. S1. The amino acid L‐tryptophan is the intrinsic precursor, which is converted to 5‐hydroxy‐L‐tryptophan (5‐HTP) by tryptophan hydroxylase (TPH) 16. TPH requires both oxygen and the cofactor tetrahydropterin (THB, also known as BH4). 5‐HTP is decarboxylated to serotonin by 5‐hydroxy‐L‐tryptophan decarboxylase (DDC) 17, and serotonin is acetylated to *N*‐acetylserotonin by serotonin *N*‐acetyltransferase (AANAT), requiring acetyl‐coenzyme A (acetyl‐CoA) 18. Finally, *N*‐acetylserotonin *O*‐methyltransferase (ASMT) methylates *N*‐acetylserotonin to the final product melatonin 19. During this last step, the conversion to melatonin is accompanied by the co‐conversion of S‐adenosyl‐L‐methionine (SAM) to S‐adenosyl‐L‐homocysteine (SAH). SAH can then be recycled back to SAM via the S‐adenosyl‐L‐methionine cycle (SAM cycle) (Fig. 1). The SAM cycle is native and constitutively expressed in budding yeast 20.

In order to establish a functional heterologous pathway that produces melatonin de novo, we ventured to construct a melatonin producing strain that utilizes endogenous L‐tryptophan generated from glucose. Since we had concerns about the stability of 5‐HTP observed in initial experiments (data not shown), we directly constructed production strains that contained the entire pathway from L‐tryptophan to melatonin. Additionally, we introduced the BH4 biosynthesis and regeneration pathways in order to avoid the need to add chemically synthesized BH4, which is the cofactor for TPH. The pathway for BH4 biosynthesis comprises a GTP cyclohydrolase I (GCH1, *FOL2* in *S. cerevisiae*), a 6‐pyruvoyl‐tetrahydropterin synthase (PTS) and a sepiapterin reductase (SPR), and converts GTP into BH4 21, 22 (Fig. 1). Conveniently, the enzyme GCH1/Fol2 is natively present in wild‐type *S. cerevisiae*, whereas heterologous PTS and SPR were introduced to enable BH4 production in budding yeast. The enzymes 4a‐hydroxytetrahydrobiopterin dehydratase (PCBD1) and 6‐pyruvoyl‐tetrahydropterin synthase (DHPR) complete the BH4 regeneration pathway.

**Figure 2:**
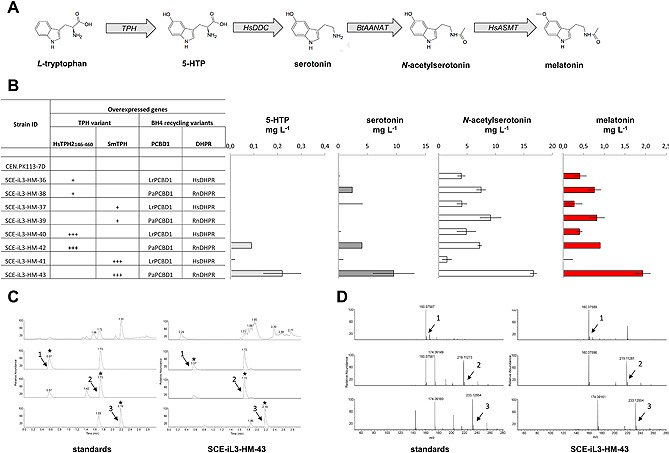
Metabolite production in yeast strains expressing the melatonin biosynthesis pathway from glucose. (**A**) Overview of the enzymatic steps for the conversion of L‐tryptophan into melatonin. (**B**) Conversion of glucose to melatonin and intermediates by recombinant *S. cerevisiae* strains. Ethanol‐extracted concentrations of indicated metabolites in the fermentation broth of cells cultivated on mineral medium as measured by LC/ESI‐MS. The strains express RnPTS, RnSPR, HsDDC, BtAANAT, HsASMT, and different combinations of *TPH*, *PCBD* and *DHPR* genes. The numbers are average values with error bars representing standard deviations for at least three individual strain isolates of one cultivation. ”+“ denotes that a single copy was integrated into the genome, ”+++“ denotes that multiple copies of the gene were integrated into the genome. (**C**) LC analysis of the standard (left), and of the bioconversion products of the representative clone 7 of strain SCE‐iL3‐HM‐43 (Ty2:: SmTPH PaPCBD1 RnDHPR RnPTS RnSPR HsDDC BtAANAT HsASMT) from glucose (right). Top: complete chromatogram. Compound 1 (serotonin), compound 2 (*N*‐acetylserotonin), and compound 3 (melatonin) have a retention time of 0.6, 1.7 and 2.2 min, respectively. Asterix indicate the main peak in the respective chromatogram. (**D**) LC‐ESI‐MS analysis of the standard and of metabolites from the production clone described in panel C in the positive mode: exact mass of compound 1 [M + H]^+^ [*m*/*z*] (160.076), compound 2 [M + H]^+^ [*m*/*z*] (219.113), compound 3 [M + H]^+^ [*m*/*z*] (233.128).

For all pathway genes, a variety of homologues were chosen based on functional conservation to the mammalian melatonin pathway enzymes, and the most promising candidates from initial functionality analysis for single substrate conversion in *E. coli* (data not shown) were chosen for strain engineering in yeast. We generated recombinant strains overexpressing *Rattus norvegicus* RnPTS, *R. norvegicus* RnSPR, one of two homologues of PCBD1 (*Lactobacillus ruminis* LrPCBD1 or *Pseudomonas aeruginosa* PaPCBD1), and one of two homologues of DHPR (*Homo sapiens* HsDHPR or *R. norvegicus* RnDHPR), thereby providing the BH4 biosynthesis and regeneration capability. Genes encoding either a double truncated version of *H. sapiens* HsTPH2 (Q8IWU9) 23, or *Schistosoma mansoni* SmTPH 24 were introduced to enable the conversion of L‐tryptophan to 5‐HTP. Here, truncated HsTPH_146‐460_ lacking both the N‐ and the C‐terminus regulatory regions was used in order to increase heterologous expression and enhance protein stability 25. Subsequently, the downstream pathway genes for producing melatonin from 5‐HTP were integrated, i.e. *H. sapiens* HsDDC, *Bos taurus* BtAANAT, and *H. sapiens* HsASMT (Fig. 2A). The derived strains were analyzed after small‐scale cultivation in mineral medium with glucose as sole carbon source. The cells produced substantially 0.3–0.8 mg L^−1^ of melatonin, 0.0–2.4 mg L^−1^ of serotonin, and 4.0–9.1 mg L^−1^ of *N*‐acetylserotonin (Fig. 2B; Supporting information, Table S5). None of these three compounds were detected in the reference non‐engineered yeast strain. Having confirmed the functionality of the de novo biosynthetic pathway, we ventured to improve the metabolic flux towards melatonin by optimizing gene expression.

### 3.2 Improved production of melatonin by higher expression of two flux controlling enzymes

Next we investigated whether improved overexpression of TPH is beneficial in order to boost the flux towards melatonin. To analyze this, we used an integrative vector that causes multi‐copy integration into Ty retrotransposon sites. Integration of the rate‐limiting enzyme into yeast Ty sites has previously been shown to be beneficial for production of 3‐hydroxypropionic acid 26. Yeast retrotransposons are a family of transposable elements that are dispersed throughout the eukaryotic genome in high numbers 27, 28, and therefore represent attractive target sites for multi‐copy integration. Accordingly, we introduced the genes coding SmTPH and HsTPH2_146‐460_ under the control of the strong P_PGK1_ promoter in multi‐copy via expression vectors targeting yeast retrotransposon Ty2 sites. We investigated TPH copy number and mRNA expression levels by QPCR and RT‐QPCR, and could confirm that there are more copies integrated, and most importantly that expression levels are substantially increased compared to single integration (Supporting information, Fig. S3, Table S6). The derived strains were tested for melatonin production after small‐scale cultivation in mineral medium. The cells with HsTPH2_146‐460_ overexpressed from Ty2 sites did not show any significant improvement of production, we measured melatonin at 0.4–0.9 mg L^−1^, and *N*‐acetylserotonin at 4.9–7.2 mg L^−1^ (Fig. 2B; Supporting information, Table S5). Interestingly, we observed a clear effect of choice of BH4 recycling pathway on production performance. Strains carrying PaPCBD1/RnDHPR produced five‐fold more melatonin than those carrying the LrPCBD1/HsDHPR homologues.

**Figure 3:**
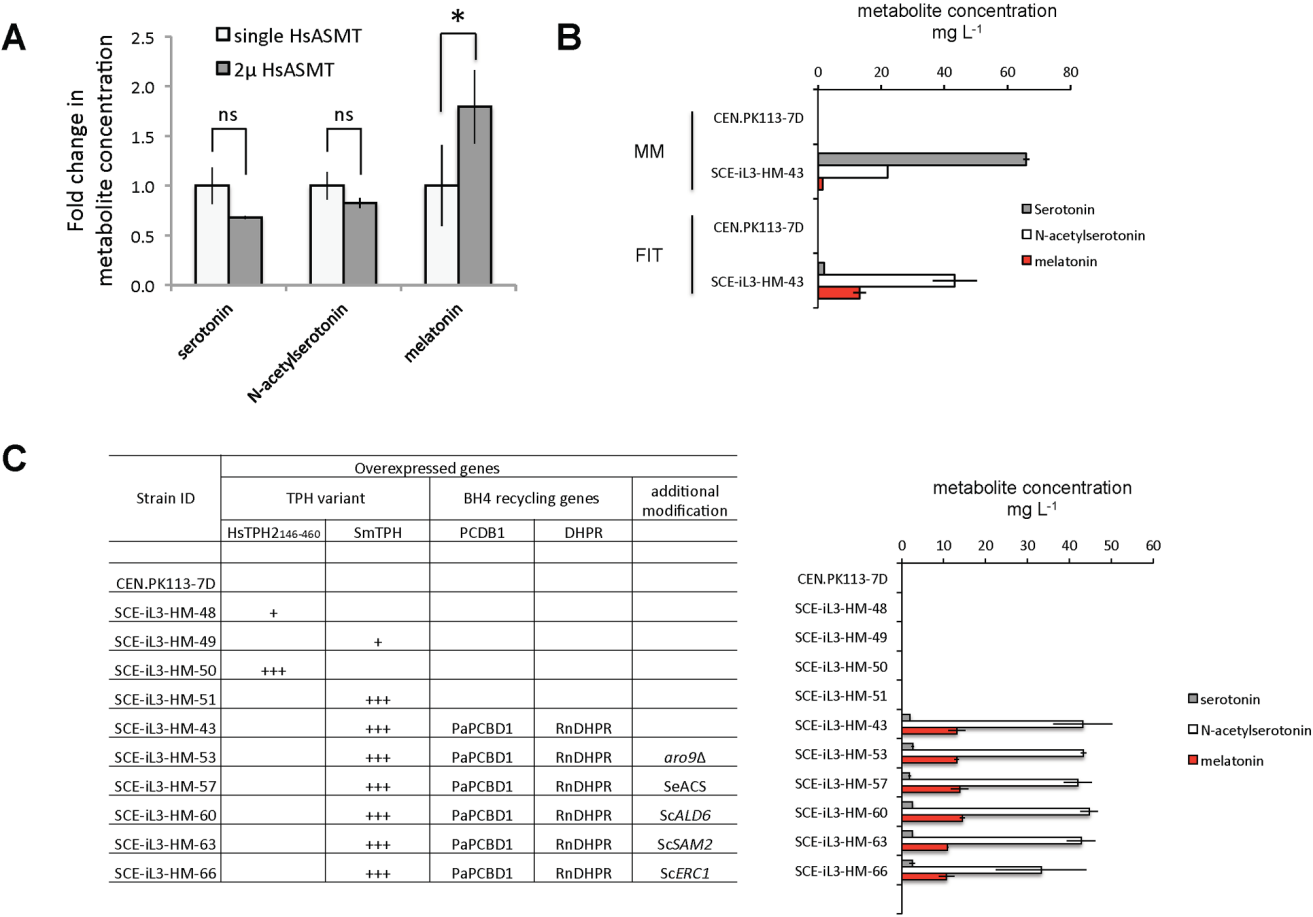
Optimization of metabolite production in yeast strains expressing the melatonin biosynthesis pathway. (**A**) Effect of introducing the episomal HsASMT plasmid on melatonin production. Strains SCE‐iL3‐HM‐44 and SCE‐iL3‐HM‐45 (Ty2:: SmTPH PaPCBD1 RnDHPR RnPTS RnSPR HsDDC BtAANAT HsASMT, transformed with pCfB3337 [pESC‐HsASMT, 2µ]), which are derived from two independent clones of SCE‐iL3‐HM‐43, were cultured in mineral medium, the supernatant extracted with ethanol, and analyzed by LC‐ESI‐MS. Measured concentrations were normalized to metabolites produced by SCE‐iL3‐HM‐43, and error bars representing standard deviations calculated from biological triplicates of one cultivation. Statistical significance of changes in production is indicated as ns (not significant) or asterix (*, significant). (**B**) Production titers of the best producing melatonin strain in different cultivation media. The non‐producing strain CEN.PK 113‐7D and the best producing strain SCE‐iL3‐HM‐43 that carries genes for overexpressing RnPTS, RnSPR, PaPCBD1, RnDHPR, HsDDC, BtAANAT, HsASMT, and Ty2:: SmTPH were cultivated in mineral medium (MM) or in FIT medium, subjected to ethanol extraction of the total cell suspension, and analyzed by LC‐ESI‐MS. The average and error bars representing standard deviations were calculated from biological duplicates of one cultivation. (**C**) Production titers of melatonin producing strains with additional genomic modifications. Strains SCE‐iL3‐HM‐48, 49, 50 and 51 carry genes for overexpressing HsDDC, BtAANAT, HsASMT, and either HsTPH_146‐460_ or SmTPH in single copy (+) or at Ty2 sites (+++), but lack all four BH4 pathway genes. Strains SCE‐iL3‐HM‐53, 57, 60, 63 and 66 are offspring of SCE‐iL3‐HM‐43 clone 61, and carry genes for overexpressing RnPTS, RnSPR, PaPCBD1, RnDHPR, HsDDC, BtAANAT, HsASMT, and Ty2:: SmTPH. In addition, either Sc*ARO9* is deleted, or one of the following genes is overexpressed: SeACS, Sc*ALD6*, Sc*SAM2*, or Sc*ERC1*. Cells were cultured in FIT media, total cell suspension extracted with ethanol, and metabolites measured by LC‐ESI‐MS. Error bars represent standard deviations, and were calculated based on biological triplicates of one cultivation.

Importantly, we achieved significantly higher titers of melatonin at 1.9 mg L^−1^, and highly increased titer of *N*‐acetylserotonin at 16.7 mg L^−1^ when overexpressing SmTPH from Ty2 sites (Ty2::SmTPH) in the presence of PaPCBD1/RnDHPR (Fig. 2B–2D; and Supporting information, Table S5), and could obtain high clonal reproducibility with regards to metabolite production (Supporting information, Fig. S2). This represents an approximate two‐fold increase in melatonin production in comparison to HsTPH2_146‐460_, indicating that *S. mansoni* TPH clearly outperforms *H. sapiens* TPH. Regarding the expression of SmTPH from Ty2 sites, we tested three independent clones and observed variable copy numbers of 2, 3 and 11. However, RT‐QPCR revealed that mRNA expression levels were increased to similar high levels of 13.6 ± 1.6 relative to *S. cerevisiae* Sc*ACT1* (Supporting information, Fig. S3, Table S6). Interestingly, the choice of BH4 recycling pathway had an even more pronounced impact on melatonin production with SmTPH overexpressed from Ty2 sites, as we observed an approximate 64‐fold increase in melatonin production when PaPCBD1/RnDHPR were integrated compared to LrPCBD1/HsDHPR (Fig. 2B; Supporting information, Table S5). We can therefore conclude that the BH4 recycling pathway with PaPCBD1 and RnDHPR is beneficial for melatonin production. In order to verify that the BH4 recycling pathway is essential for melatonin production, we constructed strains that contain the complete pathway including the TPH variants in single or multi‐copy, but lack PTS, SPR, PCBD1 and DHPR. Indeed, we did not observe any production in these strains (Fig. 3C). For future improvement, the investigation of other variants of BH4 cofactor regeneration enzymes, optimizing protein expression, or different promoter choice might be of interest.

Interestingly, we consistently observed about 10‐fold elevated levels of *N*‐acetylserotonin compared to melatonin in all melatonin‐producing strains (Fig. 2B; Supporting information, Table S5). We therefore analyzed whether we could enhance conversion by increasing the expression of HsASMT. To test this hypothesis, we introduced an episomal high‐copy (2µ) plasmid bearing HsASMT into two independent clones of the melatonin production strain that overexpress SmTPH from Ty2 sites. We observed a 1.5‐fold decrease of serotonin and a 1.2‐fold decrease of *N*‐acetylserotonin. However, both were not significantly different to the control. Importantly, we found a significant 1.8‐fold increase of melatonin when HsASMT was overexpressed from the high‐copy plasmid (*p* = 0.0202) (Fig. 3A), supporting the notion that the pathway flux is pushed towards product formation. Future research might further focus on balancing other enzymatic steps in this heterologous pathway. In addition, we investigated whether we could improve conversion of *N*‐acetylserotonin by enriching S‐adenosyl‐L‐methionine (SAM) in the cell in order to shift the ASMT reaction equilibrium towards the product. We cultured biological triplicates of all eight melatonin‐producing strains in media supplemented with 500 mg L^−1^ methionine, the precursor of SAM. However, we did not observe any significant improvement of production (data not shown). This might be due to the presence of feedback inhibiting or other regulatory processes in the cell. As an alternative, we conducted genomic engineering approaches in order to increase SAM supply. In *S. cerevisiae,* there are two isogenes, *SAM1* and *SAM2*, encoding SAM synthetase. ScS*AM2* was chosen for overexpression since it has been shown that the expression of the Sc*SAM2* gene increases during cell growth, and overrides the repressive effect of SAM 29. We also overexpressed the *S. cerevisiae* ethionine resistance conferring gene Sc*ERC1*, since it was reported that overexpressing Sc*ERC1* improves the accumulation of SAM in yeast 30. We introduced these genes into the best producing strain SCE‐iL3‐HM‐43 (carrying RnPTS, RnSPR, PaPCBD1, RnDHPR, HsDDC, BtAANAT, HsASMT and Ty2::SmTPH), cultivated independent clones in mineral or FIT medium, and performed ethanol extraction of the total cell suspension, as opposed to the extraction of supernatant in previous experiments. This different extraction method of the parent strain cultured in mineral medium resulted in increased titers of the intermediate metabolites serotonin at 65.9 ± 1.0 mg L^−1^, and *N*‐acetylserotonin at 22.1 ± 0.2 mg L^−1^. However, melatonin production was not increased and remained at 1.4 ± 0.3 mg L^−1^ (Fig. 3B; Supporting information, Table S7). Strikingly, we achieved highly decreased titers of serotonin at 2.0 ± 0.0 mg L^−1^, strongly increased titers of *N*‐acetylserotonin at 43.3 ± 7.1 mg L^−1^, and significantly higher titers of melatonin at 13.2 ± 2.1 mg L^−1^ in the parent strain when cultured in FIT medium, which represents a strong shift toward product formation (Fig. 3B; Supporting information, Table S7). Meanwhile, overexpression of Sc*SAM2* or Sc*ERC1* was not beneficial for melatonin production, as we observed no significant changes compared to parent strain (Fig. 3C; Supporting information, Table S8). Supplementing these strains with methionine decreased metabolite production even more (data not shown). We conclude that improving melatonin titers by metabolic engineering of the SAM cofactor supply is not as straightforward as anticipated. This is however very worthwhile investigating in the future, since the metabolite profile in FIT medium shows high accumulation of *N*‐acetylserotonin, and therefore the ASMT reaction seems to be the major bottleneck.

### 3.3 Further strategies for improving production titers of melatonin

Furthermore, we investigated whether increased precursor L‐tryptophan availability is beneficial for melatonin production. There are a number of tryptophan overproducers available in *E. coli* 31 or cereal crops 32. One might consider investigating these platform strains as alternative chassis for melatonin production in future research. In any case, aromatic amino acid pools are very tightly regulated in yeast *S. cerevisiae*, and to the best of our knowledge, there is no yeast tryptophan overproducer available yet. We therefore analyzed melatonin production in media supplemented with tryptophan. However, this approach did not lead to any measurable improvement (data not shown). We then ventured to analyze whether the deletion of the *S. cerevisiae* Sc*ARO9* gene might be beneficial for melatonin production, since we observed reduced degradation of 5‐HTP in initial single enzymatic conversion studies 33. Aro9 is an aromatic aminotransferase II and catalyzes the first step of tryptophan, phenylalanine, and tyrosine catabolism 34. We deleted Sc*ARO9* in the best producing strain SCE‐iL3‐HM‐43, cultivated the clones in FIT or mineral medium, and measured production. We did not observe any significant change in melatonin levels (Fig. 3C; Supporting information, Table S8). Possibly, the DDC reaction is efficient enough to compete with the 5‐HTP degradation pathways and redirects the flux towards serotonin production. Therefore, deleting Sc*ARO9* has no effect on overall production.

Alternatively, increasing acetyl‐CoA levels might improve titers of melatonin, since it is consumed during the acetylation of serotonin by AANAT 18. Therefore, a higher acetyl‐CoA/CoA ratio could favor product generation. Feeding carbon sources such as glucose improves acetyl‐CoA availability in the cell, but there are many competing metabolic pathways consuming acetyl‐CoA. Limiting oxygen can inhibit a subset of these competing pathways and thereby increase acetyl‐CoA supply 35. However, we did not further investigate this strategy, since the upstream conversion of L‐tryptophan to 5‐HTP requires oxygen, and is probably not functional under oxygen limiting conditions. Therefore, we ventured to improve the supply of acetyl‐CoA, by overexpressing the cytosolic acetaldehyde dehydrogenase Sc*ALD6* of* S. cerevisiae* or acetyl‐CoA synthase SeACS of *Salmonella enterica* 36. We introduced these modifications into the best producer SCE‐iL3‐HM‐43, cultivated the clones in FIT or mineral medium, and observed a minor increase in melatonin production by 5% (SeACS) or 10% (Sc*ALD6*) (Fig. 3C; Supporting information, Table S8). However, these changes are not significant. Nevertheless, we were confident to have obtained an improved best producing strain overexpressing Sc*ALD6* (SCE‐iL3‐HM‐60), since it produces melatonin with very low clonal variation at 14.5 ± 0.6 mg L^−1^. Lastly, increasing NADH and NADPH pools might be of interest in future investigations for improving titers of melatonin, since they are consumed during the acetylation step by AANAT 18 or during the BH4 biosynthesis and regeneration pathways, respectively.

## 4 Concluding remarks

In contrast to biological tissue extraction and chemical synthesis, production of active biological ingredients using metabolically engineered microorganisms fermenting renewable feedstocks is an attractive process. In this study, we have assembled a recombinant melatonin pathway in *S. cerevisiae* and demonstrated de novo production of melatonin and its related compounds from glucose for the first time. We have identified the ASMT reaction as the major bottleneck, and gene expression optimization was proven to be beneficial. Overall, our initial exploration is providing a promising basis for future improvements and investigations.

## Supporting information

As a service to our authors and readers, this journal provides supporting information supplied by the authors. Such materials are peer reviewed and may be re‐organized for online delivery, but are not copy‐edited or typeset. Technical support issues arising from supporting information (other than missing files) should be addressed to the authors.

Supporting Information
