# Supplementary material for: Glucose‐based microbial production of the hormone melatonin in yeast Saccharomyces cerevisiae
Source: Biotechnol J. 2016 Jan 25;11(5):717–24. doi: 10.1002/biot.201500143 (PMC5066760; doi:10.1002/biot.201500143)
Supplement: Supplementary file 1 — Supporting Information [file BIOT-11-717-s001.pdf]

Biotechnology
Journal

Supporting Information for DOI 10.1002/biot.201500143

Glucose-based microbial production of the hormone melatonin in yeast *Saccharomyces cerevisiae*

Susanne M. Germann, Simo A. Baallal Jacobsen, Konstantin Schneider, Scott J. Harrison, Niels B. Jensen, Xiao Chen, Steen G. Stahlhut, Irina Borodina, Hao Luo, Jiangfeng Zhu, Jérôme Maury, Jochen Forster

© 2015 Wiley-VCH Verlag GmbH & Co. KGaA, Weinheim

Supporting Information

Production of the mammalian hormone melatonin from glucose in *Saccharomyces cerevisiae*.

Susanne M. Germann*, Simo A. Baallal Jacobsen*, Konstantin Schneider, Scott J. Harrison, Niels B. Jensen, Xiao Chen, Steen G. Stahlhut, Irina Borodina, Hao Luo, Jiangfeng Zhu, Jérôme Maury, Jochen Forster§

The Novo Nordisk Foundation Center for Biosustainability, Technical University of Denmark, Kogle Allé 6, 2970 Hørsholm, Denmark

* These authors contributed equally to this work

§ Corresponding author: Jochen Forster. E-mail: jfor@biosustain.dtu.dk

Abbreviations

Enzymes and their corresponding EC numbers.

Abbreviation	Enzyme	EC #
AANAT	serotonin acetyltransferase	EC 2.3.1.87 or EC 2.3.1.5
ACS	acetyl-CoA synthase	EC 2.3.1.169
ALD6	aldehyde dehydrogenase	EC 1.2.1.4
ARO9	aromatic aminotransferase II	EC 2.6.1.57
ASMT	acetylserotonin O-methyltransferase	EC 2.1.1.4
DDC	5-hydroxy-*L*-tryptophan decarboxylase, a.k.a. dopa decarboxylase (aromatic *L*-amino acid decarboxylase)	EC 4.1.1.28
DHPR	dihydropteridine reductase	EC 1.5.1.34
ERC1	ethionine resistance conferring gene	N/A
GCH1	GTP cyclohydrolase I	EC 3.5.4.16
MS	methionine synthase	EC 2.1.1.-
PCBD	pterin-4-alpha-carbinolamine dehydratase, a.k.a. 4a-hydroxytetrahydrobiopterin dehydratase	EC 4.2.1.96
PTS a.k.a. PTPS	6-pyruvoyl-tetrahydropterin synthase	EC 4.2.3.12
SAH1	*S*-adenosyl-homocysteine hydrosylase	EC 3.3.1.1
SAM2	S-adenosylmethionine synthetase	EC 2.5.1.6
SAMS	*S*-adenosyl-methionine synthetase	EC 2.5.1.6
SPR	sepiapterin reductase	EC 1.1.1.153
TPH	*L*-tryptophan hydroxylase	EC 1.14.16.4

Metabolites and their corresponding PubChem numbers.

Abbreviation	Metabolite	PubChem CID
Acetyl-CoA	acetyl coenzyme A	444493
BH4/THB	tetrahydrobiopterin	1125
DHB	dihydrobiopterin	119055
DHP	7,8-dihydroneopterin 3'-triphosphate	121885
GTP	guanosine triphosphate	6830
HTHB	4a-hydroxytetra-hydrobiopterin	129803
5-HTP	5-hydroxy-L-tryptophan	144
NADH	nicotinamide adenine dinucleotide	5893

NADPH	nicotinamide adenine dinucleotide phosphate	5884
6-PTH	6-pyruvoyltetrahydropterin	128973
SAM	*S*-adenosyl-*L*-methionine	34755
SAH	*S*-adenosyl-*L*-homocysteine	439155

Supplementary Methods

Synthetic genes for yeast expression constructs

Genes encoding a *B. taurus* arylalkylamine N-acetyltransferase BtAANAT (*GenBank: 281583*), *H. sapiens* acetylserotonin O-methyltransferase HsASMT (*GenBank:* 438), *H. sapiens* 5-hydroxy-*L*-tryptophan decarboxylyase HsDDC (*GenBank: 1644*), *H. sapiens* 6-pyruvoyl-tetrahydropterin synthase HsDHPR (*GenBank:* 5860), *R. norvegicus* 6-pyruvoyl-tetrahydropterin synthase RnDHPR (*GenBank:* 64192), *L. ruminis* pterin-4-alpha-carbinolamine dehydratase LrPCBD1 (*GenBank:* WP_003692157.1), *P. aeruginosa* pterin-4-alpha-carbinolamine dehydratase PaPCBD1 (*GenBank:* 880827), *R. norvegicus* 6-pyruvoyl-tetrahydropterin synthase RnPTPS a.k.a. RnPTS (*GenBank:* 29498), *R. norvegicus* sepiapterin reductase RnSPR (*GenBank:* 29270), a double truncated *H. sapiens* tryptophan hydroxylase $HsTPH2_{146\text{-}460}$ (Q8IWU9) (*GenBank:* 121278), and *S. mansoni* tryptophan hydroxylase SmTPH (*GenBank:* AF031034.1) were synthesized by GeneArt (Life Technologies) in versions codon-optimized for yeast *S. cerevisiae* or *E. coli* (as indicated in Table s2). The synthetic gene constructs had a general structure: **ATG**NN...NN**TGA**, where ATG is the start codon, NN...NN represents the protein coding sequence without start and stop codons, TGA (or alternatively TAA) is the stop codon. The Kozak sequence (AAAACA) upstream of the start codon for improved yeast transcription was introduced via the gene BioBrick primers (Table s1).

Cloning of yeast expression constructs

The gene fragments (BioBricks) carrying the genes and correct overhangs for USER-cloning were generated by PCR amplification using primers and templates as indicated in Table s2. The PCR mix contained: The PCR mix contained: 18 µl water, 10 µl HF phusion buffer (5x, BioLab), 5 µl 2mM dNTP, 2 µl PfuX7 polymerase [1], 2.5 µl forward primer (10 µM), 2.5 µl reverse primer (10 µM), and 1 µl DNA template. The cycling program was: 95°C for 2 min, 30 cycles of [95°C for 10 sec, 52°C for 20 sec, 68°C for (1min/kb)], 68°C for 5 min, pause at 10°C. The gene fragments were resolved on 1% agarose gel containing SYBR®-SAFE (Invitrogen) and purified using NucleoSpin® Gel and PCR Clean-up kit (Macherey-Nagel). The promoter fragments were also generated by PCR followed by DNA purification (Table s2). The terminators were already present on the yeast vectors. The expression plasmids were created by USER-cloning as described previously using *E. coli* strain DH5alpha [2]. The clones with correct inserts were identified by colony PCR and the plasmids of 4 clones/transformation were

isolated from overnight *E. coli* cultures and confirmed by sequencing (Eurofins). The expression plasmids are listed in Table s3, and the primers used are listed in Table s1. Construction of plasmids pCfB2772 and pCfB2773 was mainly performed on the Hamilton Vantage Cloning Robot.

In the course of this study, plasmid pCfB998 was found to have acquired the point mutation G163C in BtAANAT. Since this caused the altered translated protein BtAANAT-A55P, we fixed this mutation by site-directed mutagenesis to generate pCfB2628 using primers PR-8518/PR-8519 and the QuikChange II XL Site-Directed Mutagenesis Kit (Agilent Technologies) according to manufacturer's instructions.

For subcloning HsASMT into a high-copy 2μ vector, we created a terminator-gene1-promoter1 BioBrick with primers PR-6/PR-11055 on template pCfB1252, and USER cloned this BioBrick into USER-prepared pESC-LEU-USER cloning site (SEQ ID NO: 15).

Construction of yeast production strains

The yeast expression plasmids were transformed into *S. cerevisiae* cells using the lithium acetate transformation protocol [3]. Prior to transformation, integrative vectors were digested by *Not*I and column-purified (Nucelospin Gel and PCR cleanup kit, Macherey Nagel). Approximately 1 μg DNA (single integrative) or 1.5 μg (multi-copy integrative, Ty2) was transformed into competent yeast cells. The cells were selected on drop-out agar medium, and correct integration at the specific genomic loci was verified by colony PCR. Cre-loxP-mediated selection marker loop out was performed as described previously [2]. After loop out, colony PCR was performed to confirm the retained presence of the integrated markerless genes using the same primer sets for cloning the initial gene BioBricks (Table s1). The resulting yeast strains are listed in Table s4.

The *aro9*Δ deletion strain was constructed by amplifying the KanMX-deleted ORF from genomic DNA of the *aro9*Δ::KanMX deletion strain of the YKO *MAT*a Strain Collection (open biosystems), using primers PR-13241/PR-13242, and transforming 1 μg of this deletion cassette into yeast. Correct integration was confirmed by colony PCR with primers PR-477/PR-13441.

The plasmids and strains described in this work will be readily provided on request to the corresponding author.

Metabolite analysis by LC-ESI-MS

LC-ESI-MS data was collected on OrbiTrap Fusion High Resolution Mass Spectrometer system coupled with an Ultimate 3000 UHPLC pump (Thermo, San Jose Ca). Samples were held in the autosampler at a temperature of 10.0°C during the analysis. 1µL Injections of the sample were made onto a Thermo HyperSil Gold PFP HPLC column, with a 3 um particle size, 2.1 mm i.d. and 150 mm long. The column was held at a temperature of 35.0°C. The solvent system used was Solvent A "Water with 0.1% formic acid" and Solvent B "Acetonitrile with 0.1% formic ". The Flow Rate was 1.000 ml/min with an Initial Solvent composition of %A = 95, %B = 5 held until 0.50 min, the solvent composition was then changed following a Linear Gradient until it reached %A = 70.0 and %B = 30.0 at 1.50 min. The solvent composition was then changed following a Linear Gradient until it reached %A = 5.0 and %B = 95.0 at 2.00 min This was held until 2.50 min when the solvent was returned to the initial conditions and the column was re-equilibrated until 3.00 min. The first 0.25 min of the run was diverted to waste using the divert valve, following which the column eluent flowed directly into the Heated ESI probe of the MS which was held at 325°C and a voltage of 3500 V. Data was collected in positive ion mode over the mass range 50 to 1000 m/z at a resolution of 15.000. The other MS settings were as follows, Sheath Gas Flow Rate of 60 units, Cone Gas Flow Rate of 20 units Cone Temp was 275°C.

Cell harvest, DNA purification and total RNA extraction, and reverse transcription

Strains were grown overnight at 30°C in SC-ura media. The next day, 2.4 x 10^8 cells were harvested for DNA extraction, and kept at -20°C until further processing. For RNA extraction, the remaining cultures were diluted to OD_{600} = 0.2 and grown to mid-log phase (OD_{600} ~ 0.5). Then 3.6 x 10^8 cells were harvested, snap frozen in liquid nitrogen, and kept at -80°C until further processing. Purified genomic DNA was prepared as described before [4]. Total RNA extraction was performed using the PureLink® RNA Mini Kit (Ambion, Life Technologies). Cells were lysed enzymatically for 1h at 30°C with Zymolase® 100T (amsbio) according to manufacturers' instructions. RNA samples were then treated with DNaseI for 30 min at 37°C, and DNaseI subsequently inactivated. Isolated total RNA integrity was verified by agarose gel electrophoresis and SYBR® Safe DNA gel staining (Thermo Fisher Scientific) of samples denatured by formamide (Sigma), and by an average UV absorbance ratio A_{260}:A_{280} of 2.14 (range 2.1 – 2.2). 500 ng total RNA was reverse transcribed with the First Strand cDNA Synthesis Kit (Thermo Scientific) using random hexamer primers according to manufacturers' instructions, and included a non-template control (NTC), the manufacturer's positive control (PC), and

control reactions not containing reverse transcriptase (-RT) to verify the absence of genomic DNA.

Quantitative real-time PCR (QPCR) and reverse-transcription quantitative real-time PCR (RT-QPCR)

The copy numbers of the $HsTPH2_{146\text{-}460}$ and the SmTPH genes were determined by quantitative real-time PCR (QPCR) analysis, and mRNA levels by reverse-transcription quantitative real-time PCR (RT-QPCR) using primer pair PR-14437/PR-14438 or PR-13245/PR-13256, respectively. All values were normalized to the reference gene *ACT1*, amplified with primers PR-14439/PR-14440. All QPCR and RT-QPCR reactions were performed in the real-time thermal cycler Mx3005P (Stratagene, Agilent Technologies) using the SYBR® Select Master Mix (Life Technologies) with 0.08µl DNA or 2µl cDNA as template. For standard curves, DNA/cDNA of a strain with a single copy of the respective TPH was used ($HsTPH2_{146\text{-}460}$: SCE-iL3-HM-48_1; SmTPH: SCE-iL3-HM-49_1). PCR plates were sealed with MicroAmp® Optical Adhesive Film (Life Technologies). The following PCR cycling conditions were used: (i) UDG activation (2 min at 50°C); (ii) AmpliTaq® DNA polymerase, UP activation (2 min at 95°C); (iii) amplification and quantification program repeated 40 times (15 sec at 95°C; 30 sec at 54°C; 1 min at 72°C with a single fluorescence measurement); (iv) melting curve program (1 min at 95°C; 54-95°C with a continuous fluorescence measurement); (v) cooling program down to 12°C. Calibration curves, efficiencies, correlation coefficients, and threshold cycles (C_T) for the amplification curves are available on request. Data was analyzed using the MxPro QPCR Software for Mx3000P and Mx3005P QPCR Systems (Stratagene, Agilent Technologies) and Microsoft Excel. Specificity was confirmed empirically by gel electrophoresis and melting curve profiles. Non-template controls (NTCs) were performed to detect PCR contaminations and primer dimers. All reactions were measured in triplicates.

Supplementary Figures

Figure s1. Production pathway for *de novo* biosynthesis of melatonin in *S. cerevisiae* and its connection to the intrinsic yeast metabolism. Central yeast metabolic pathways, substrate synthesis, energy metabolism, transporters and the interface to the heterologous pathways are shown.

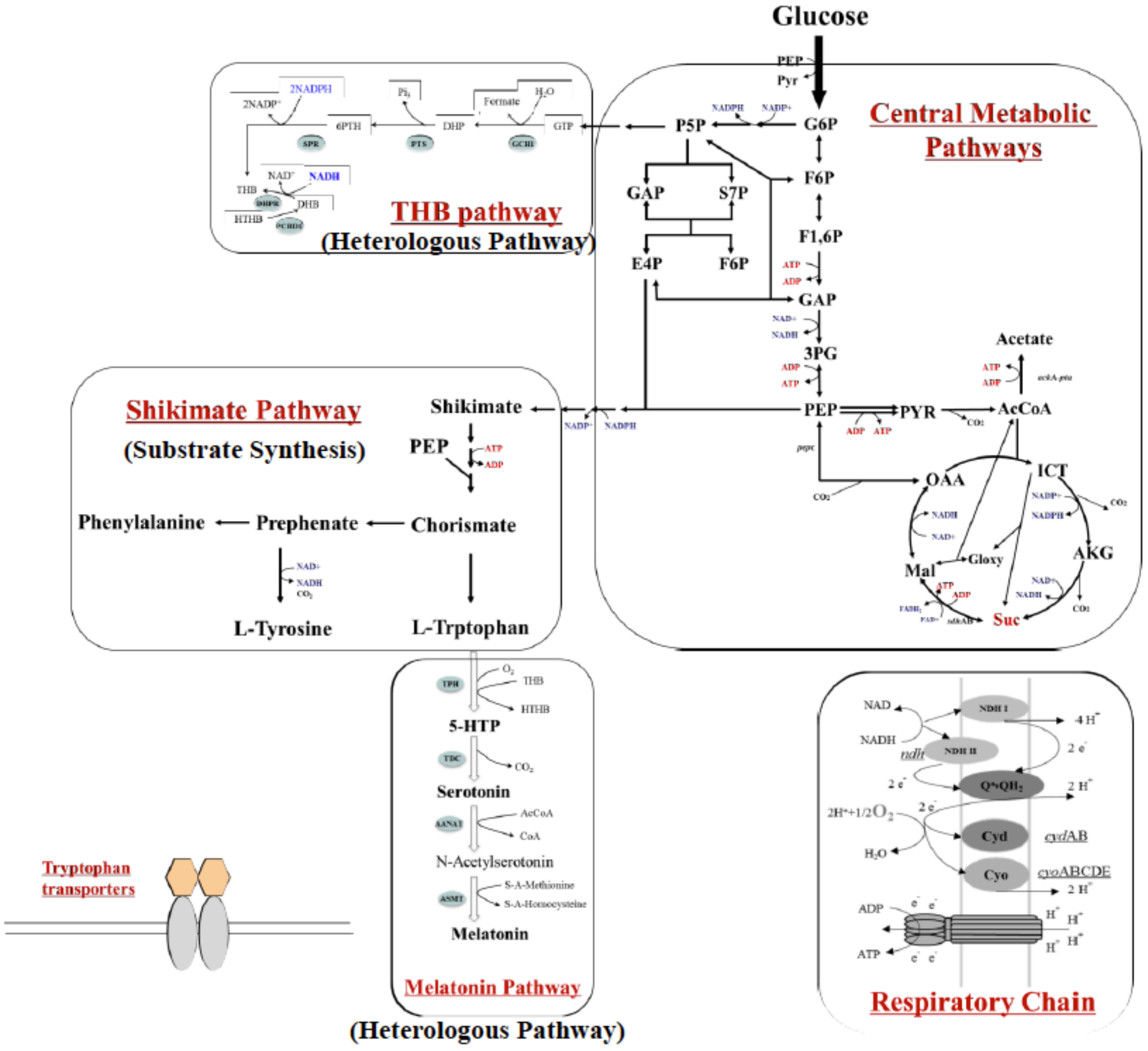

Figure s2. Clonal reproducibility of metabolite production in yeast strains expressing the melatonin biosynthesis pathway from glucose. (A) HPLC analysis of bioconversion products of individual clones of *S. cerevisiae* strain SCE-iL3-HM-43 (SmTPH PaPCBD1 RnDHPR RnPTS RnSPR HsDDC BtAANAT HsASMT). (i) melatonin standard (control), (ii) clone 7, (iii) clone 12, (iv) clone 13, (v) clone 17, (vi) clone 22, and (vii) clone 40. Compound **3** (melatonin) has a retention time of 2.2 min. (B) LC/ESI-MS analysis of metabolites from the *S. cerevisiae* clones described in panel A in the positive mode: exact mass of compound **3** (melatonin) [M + H]+ [m/z] (233.128).

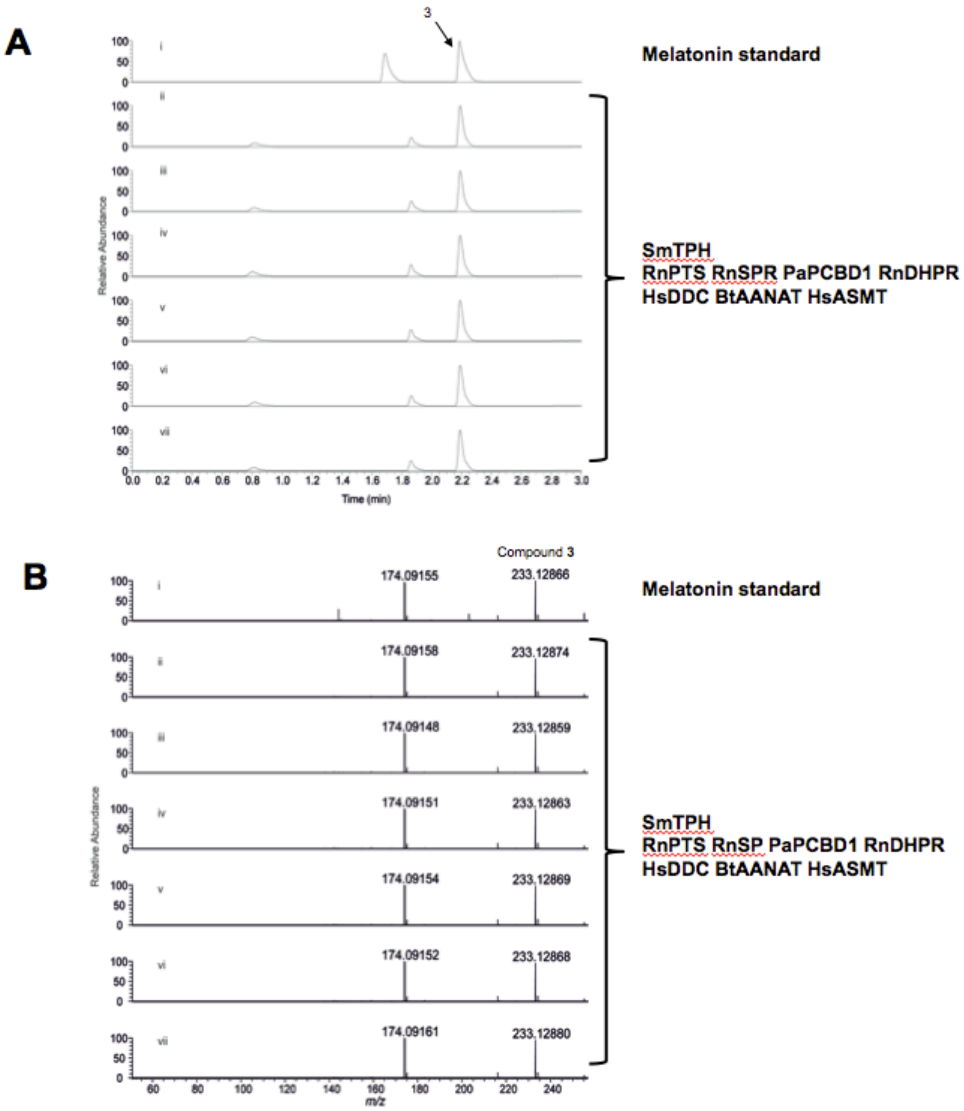

Figure s3. Analysis of SmTPH and HsTPH$_{146\text{-}460}$ copy number and mRNA levels in the respective production strains. Strains were cultured in SC-ura and harvested for DNA or RNA extraction, and subsequently QPCR or RT-QPCR was performed with primer pairs for SmTPH, HsTPH$_{146\text{-}460}$, and Sc*ACT1* as reference. Relative SmTPH and HsTPH$_{146\text{-}460}$ copy number and mRNA levels were found by normalizing to Sc*ACT1*. All reactions were performed in triplicate, error bars represent SD. Numerical values are shown in table s6.

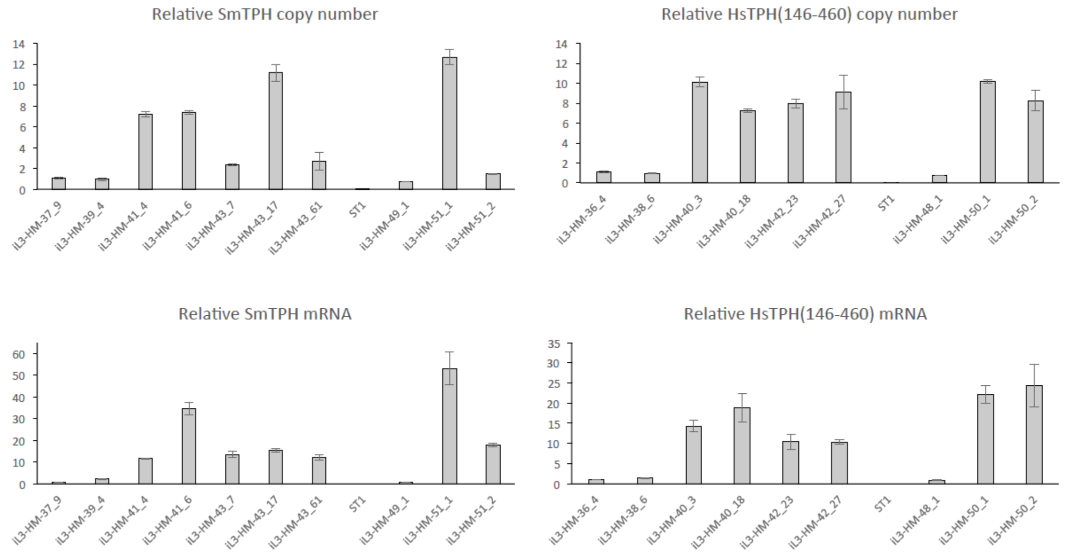

Supplementary Tables

Table s1: Primer sequences. Sequences of primers (5´ to 3´) used in this study. Overhangs used for USER cloning are underlined.

**Primer ID**	**Primer name**	**Primer sequence, 5´ to 3´**
PR-5	PTEF1-fw	ACCTGCAC**U** TTGTAATTAAAACTTAG
PR-6	PTEF1-rv	CACGCGA**U** GCACACACCATAGCTTC
PR-7	PPGK1-fw	CGTGCGA**U** GGAAGTACCTTCAAAGA
PR-8	PPGK1-rv	ATGACAGA**U** TTGTTTTATATTTGTTG
PR-278	pTDH3_fw	CGTGCGA**U** ATAAAAAACACGCTTTTTCAGTTCG
PR-279	pTDH3_rv	ATGACAGA**U** TTTGTTTGTTTATGTGTGTTTATTC
PR-394	RnSPR-fw	ATCTGTCA**U** AAAACAATGGAAGGAGGCAGGCTAG
PR-389	RnSPR-rv	CACGCGA**U** TTAAATGTCATAGAAGTCCACGTG
PR-393	RnPTS-fw	AGTGCAGG**U** AAAACAATGAACGCGGCGGTTGG
PR-350	RnPTS-rv	CGTGCGA**U** TTATTCTCCTTTGTAGACCACAAT
PR-477	KanMX_2/3_START_rv	AGTGACGACTGAATCCGGTG
PR-738	ALD6_U2_fw	ATCTGTCA**U** AAAACAATGACTAAGCTACACTTTGACAC
PR-739	ALD6_U2_rv	CACGCGA**U** TCACAACTTAATTCTGACAGCTTTTAC
PR-1759	HsDDC-rv	CGTGCGA**U** TTATTCACGTTCGGCACGCAGCAC
PR-1760	HsDDC-fw	AGTGCAGG**U** AAAACAATGAATGCAAGCGAATTTCGTCG
PR-1761	BtAANAT-fw	ATCTGTCA**U** AAAACAATGAGCACCCCGAGCATTCATTG
PR-1762	BtAANAT-rv	CACGCGA**U** TTAACGATCGCTATTACGACGCAGTG
PR-1764	GgASMT-fw	AGTGCAGG**U** AAAACAATGGATAGCACCGAAGATCTGG
PR-1763	GgASMT-rv	CGTGCGA**U** TTATTTACGACCCAGAACTGCATC
PR-2148	PaPCBD1-fw	AGTGCAGG**U** AAAACAATGACTGCTTTGACTCAAGC
PR-2149	PaPCBD1-rv	CGTGCGA**U** TTACTTTCTACCTTCAGCAG
PR-2150	LrPCBD1-fw	AGTGCAGG**U** AAAACAATGGTCAAGTTGTTCCCATC
PR-2151	LrPCBD1-rv	CGTGCGA**U** TCAAATTCTGGCATCTTGAATTTC
PR-2152	HsDHPR-fw	ATCTGTCA**U** AAAACAATGGCTGCTGCTGC
PR-2153	HsDHPR-rv	CACGCGA**U** TTAGAAGTAAGCTGGAGTC
PR-2154	RatDHPRyeast-fw	ATCTGTCA**U** AAAACAATGGCTGCTTCTGG
PR-2155	RatDHPRyeast-rv	CACGCGA**U** TTAGAAGTAAGCTGGAGTCAATTC
PR-2254	HsASMT-fw	AGTGCAGG**U** AAAACAATGGGTAGCAGCGAAGATC
PR-2255	HsASMT-rv	CGTGCGA**U** TTATTTACGTGCCAGGATTGCATC
PR-8502	SmTPH-fw	ATCTGTCA**U** AAAACAATGATTAGCACCGAAAGCG
PR-8503	SmTPH-rv	CACGCGA**U** TTAGCTGCTGCGATTTTCG
PR-8504	HsTPH2-(146-460)-fw	ATCTGTCA**U** AAAACAATGGAACTGGAAGATGTTCCG
PR-8505	HsTPH2-(146-460)-rv	CACGCGA**U** TTAGGTATCTTTCAGGATCTCGATG
PR-8518	BtAANAT correction-fw	GTTTTTGAAATTGAACGTGAAGCATTTATTAGCGTGAGCGG
PR-8519	BtAANAT correction-rv	CCGCTCACGCTAATAAATGCTTCACGTTCAATTTCAAAAAC
PR-11055	TADH1_U1_fw	CGTGCGA**U** GAGCGACCTCATGCTATACC
PR-13235	SeACS_U2_F	ATCTGTCA**U** AAAACAATGTCACAAACACAC

Primer ID	Primer name	Primer sequence, 5´ to 3´
PR-13236	SeACS_U2_R	CACGCGA**U** TCATGATGGCATAGCAATAG
PR- 13237	ScSAM2_U2_F	ATCTGTCA**U** AAAACAATGTCCAAGAGCAAAACTTTCTTATTTAC
PR- 13238	ScSAM2_U2_R	CACGCGA**U** TTAAAATTCCAATTTCTTTGGTTTTTCCC
PR- 13239	ScERC1_U2_F	ATCTGTCA**U** AAAACAATGTCTAAACAATTTAGTCATACCACCAAC
PR- 13240	ScERC1_U2_R	CACGCGA**U** CTAGTTATACCCAACCATAAGCCG
PR- 13241	ARO9_up_F	GGTAGATAAGAGAGCGGAGCACGTGG
PR- 13242	ARO9_down_R	GGAGAGAACAAATGGATAAGTTGCGTTTCCTC
PR- 13441	ARO9_dw_seq_R	GGAAAACGCAAGTGGATAAAGGGGTGGG
PR-13245	SmTPH_int_F	GAGCCATCATCAGAACGTTCAAGAAATG
PR-13246	SmTPH_int_R	CAATACACGGAACTTCAACATGAACCAGC
PR-14437	HsTPH_int_F2	CGCTGCTGACCAAATATTGTGG
PR-14438	HsTPH_int_R2	CAGCAGCGGCACATGACC
PR-14439	ACT1_F1	ACTGAAGCTCCAATGAACCCTA
PR-14440	ACT1_R1	GTCCAAGGCGACGTAACATAGT

Table s2: DNA BioBricks for yeast expression plasmids. Genes and promoters (BioBricks) were PCR-amplified from the indicated templates using the corresponding forward and reverse oligos.

BioBrick name [a)]	Description	Oligo forward	Oligo reverse	Template
<-ScP$_{TEF1}$	Promoter of *TEF1* gene (*S. cerevisiae*)	PR-5	PR-6	pSP-GM1
->ScP$_{PGK1}$	Promoter of *PGK1* gene (*S. cerevisiae*)	PR-7	PR-8	pSP-GM1
<-ScP$_{TEF1}$-ScP$_{PGK1}$->	Fused promoters of *TEF1* and *PGK1* genes (*S. cerevisiae*)	PR-5	PR-8	pSP-GM1
ScP$_{TDH3}$->	Promoter of *TDH3* gene (*S. cerevisiae*)	PR-278	PR-279	Genomic DNA of CEN.PK113-7D
BtAANAT->	arylalkylamine N-acetyltransferase gene (*B. taurus*) [b)]	PR-1761	PR-1762	pCfB554, vector with cloned synthetic gene (GeneArt), SEQ ID NO: 1
HsASMT<-	acetylserotonin O-methyltransferase gene (*H. sapiens*) [b)]	PR-2254	PR-2255	pCfB560, vector with cloned synthetic gene (GeneArt), SEQ ID NO: 2
HsDDC<-	5-hydroxy-L-tryptophan decarboxy-lyase gene (*H. sapiens*) [b)]	PR-1759	PR-1760	pCfB564, vector with cloned synthetic gene (GeneArt), SEQ ID NO: 3
HsDHPR->	6-pyruvoyl-tetrahydropterin synthase gene (*H. sapiens*) [c)]	PR-2152	PR-2153	pCfB3517, vector with cloned synthetic gene (GeneArt), SEQ ID NO: 4
RnDHPR->	6-pyruvoyl-tetrahydropterin synthase gene (*R. norvegicus*) [c)]	PR-2154	PR-2155	pCfB3518, vector with cloned synthetic gene (GeneArt), SEQ ID NO: 5
LrPCBD1<-	4a-hydroxytetrahydrobiopterin dehydratase 1 gene (*L. ruminis*) [c)]	PR-2150	PR-2151	pCfB3519, vector with cloned synthetic gene (GeneArt), SEQ ID NO: 6
PaPCBD1<-	4a-hydroxytetrahydrobiopterin dehydratase gene (*P. aeruginosa*) [c)]	PR-2148	PR-2149	pCfB3520, vector with cloned synthetic gene (GeneArt), SEQ ID NO: 7
RnPTS<-	6-pyruvoyl-tetrahydropterin synthase gene (*R. norvegicus*) [c)]	PR-393	PR-350	pCfB1205, vector with cloned synthetic gene (GeneArt), SEQ ID NO: 8
RnSPR->	sepiapterin reductase gene (*R. norvegicus*) [c)]	PR-394	PR-389	pCfB1205, vector with cloned synthetic gene (GeneArt), SEQ ID NO: 9
HsTPH2$_{146-460}$->	double truncated tryptophan hydroxylase 2 gene (*H. sapiens*)	PR-8504	PR-8505	pCfB2563, vector with cloned synthetic gene (GeneArt), SEQ ID NO: 10
SmTPH->	tryptophan hydroxylase gene *(S. mansoni)*	PR-8502	PR-8503	pCfB2562, vector with cloned synthetic gene (GeneArt), SEQ ID NO: 11
ScALD6->	cytosolic acetaldehyde dehydrogenase gene *(S. cerevisiae)*	PR-738	PR-739	Genomic DNA of CEN.PK113-7D
SeACS$_{L641P}$->	acetyl-CoA synthase gene with point mutation L641P (*S. enterica)*	PR-13235	PR-13236	pIYC05
ScSAM2->	S-adenosylmethionine synthetase gene (*S. cerevisiae)*	PR-13237	PR-13238	Genomic DNA of CEN.PK113-7D
ScERC1->	ethionine resistance conferring *gene* (*S. cerevisiae)*	PR-13239	PR-13240	Genomic DNA of CEN.PK113-7D
aro9Δ::KanMX	aromatic aminotransferase II gene (*S. cerevisiae*)	PR-13241	PR-13242	Genomic DNA of *aro9*Δ::KanMX strain of YKO *MAT*a Strain Collection (open biosystems)

a) “<-“ indicates gene position 1 and “->” indicates gene position 2 as described in [2]

b) codon-optimized for *E. coli*

c) codon-optimized for *S. cerevisiae*

Table s3: List of plasmids used in this study. The plasmids were constructed by assembling parent plasmid and BioBricks. The resulting relevant gene content, integration site or replicon type and selection maker are shown.

Plasmid name	Parent plasmid, BioBricks	Gene content	Integration site/replicon	Selection marker	Reference/ Source
pESC-URA (pCfB22)	-	-	2m	KlURA3	Agilent
pESC-LEU (pCfB24)	-	-	2m	KlLEU2	Agilent
pSP-GM1 (pCfB29)	-	P_{TEF1}, P_{PGK1}	2m	URA3	[5]
pCfB255	-	USER cloning site	X-2	loxP-KlURA3	[2]
pCfB257	-	USER cloning site	X-3	loxP- KlLEU2	[2]
pCfB258	-	USER cloning site	X-4	loxP-SpHIS5	[2]
pCfB259	-	USER cloning site	XII-1	loxP-KlLEU2	[2]
pIYC05	-	P_{TEF1}::SeACS$_{L641P}$ P_{PGK1}::ScALD6	2m	HIS3	[6]
pCfB390	-	USER cloning site	XI-3	loxP-KlURA3	[2]
pCfB391	-	USER cloning site	XI-5	loxP-SpHIS5	[2]
pCfB997	pCfB259, GgASMT<-, ScP$_{TEF1}$	P_{TEF1}::GgASMT	XII-1	loxP-KlLEU2	This study
pCfB998	pCfB391, HsDDC<-, ScP$_{TEF1}$-ScP$_{PGK1}$, BtAANAT-A55P->	P_{TEF1}::HsDDC P_{PGK1}::BtAANAT$_{A55P}$	XI-5	loxP-SpHIS5	This study
pCfB1248	pCfB258, PaPCBD1<-, ScP$_{TEF1}$-ScP$_{PGK1}$, RnDHPR->	P_{TEF1}::PaPCBD1 P_{PGK1}::RnDHPR	X-4	loxP-SpHIS5	This study
pCfB1249	pCfB258, LrPCBD1<-, ScP$_{TEF1}$-ScP$_{PGK1}$, HsDHPR->	P_{TEF1}::LrPCBD1 P_{PGK1}::HsDHPR	X-4	loxP-SpHIS5	This study
pCfB1251	pCfB257, RnPTS<-, ScP$_{TEF1}$-ScP$_{PGK1}$, RnSPR->	P_{TEF1}::RnPTS P_{PGK1}::RnSPR	X-3	loxP- KlLEU2	This study
pCfB1252	pCfB259, HsASMT<-, ScP$_{TEF1}$	P_{TEF1}::HsASMT	XII-1	loxP- KlLEU2	This study
pCfB2224	-	USER cloning site	XI-2	loxP-KanMXsyn	This study
pCfB2528	pCfB390, SmTPH->, ScP$_{PGK1}$	P_{PGK1}::SmTPH	XI-3	loxP-KlURA3	This study
pCfB2529	pCfB390, HsTPH2$_{146-460}$->, ScP$_{PGK1}$	P_{PGK1}::HsTPH$_{146-460}$	XI-3	loxP-KlURA3	This study
pCfB2628	pCfB998, HsDDC<-, ScP$_{TEF1}$-ScP$_{PGK1}$, BtAANAT->	P_{TEF1}::HsDDC P_{PGK1}::BtAANAT	XI-5	loxP-SpHIS5	This study
pCfB2772	pTY2-loxP-URA3-degron, SmTPH->, ScP$_{PGK1}$	P_{PGK1}::SmTPH	TY2	loxP-KlURA3-degron	This study SEQ ID NO: 12
pCfB2773	pTY2-loxP-URA3-degron, HsTPH2$_{146-460}$->, ScP$_{PGK1}$	P_{PGK1}::HsTPH$_{146-460}$	TY2	loxP-KlURA3-degron	This study SEQ ID NO: 13
pCfB3337	pESC-LEU-USER cloning site, TADH1, HsASMT<-, ScP$_{TEF1}$	P_{TEF1}::HsASMT	2μ	NatMX	This study SEQ ID NO: 14
pCfB4149	pCfB2224, SeACS->, ScP$_{TDH3}$	P_{TDH3}::SeACS	XI-2	loxP-KanMXsyn	This study
pCfB4150	pCfB2224, ScALD6->, ScP$_{TDH3}$	P_{TDH3}::ScALD6	XI-2	loxP-KanMXsyn	This study
pCfB4151	pCfB2224, ScSAM2->, ScP$_{TDH3}$	P_{TDH3}::ScSAM2	XI-2	loxP-KanMXsyn	This study
pCfB4152	pCfB2224, ScERC1->, ScP$_{TDH3}$	P_{TDH3}::ScERC1	XI-2	loxP-KanMXsyn	This study

Table s4: List of strains used in this study. The strains were constructed by transforming plasmids into yeast chassis (parent strains).

Strain name	Parent strain (chassis)	Added plasmid	Relevant genotype	Reference/ Source
CEN.PK113-7D	-	-	*MAT*a URA3 HIS3 LEU2 TRP1 MAL2-8^c SUC2	Peter Kötter
CEN.PK102-5B	-	-	*MAT*a *ura3-52 his3 D 1 leu2-3/112* MAL2-8c SUC2 [ura$^-$ his$^-$ leu$^-$]	Peter Kötter
ST3725	BY4741	-	*MAT*a *his3Δ1 leu2Δ0 met15Δ0 ura3Δ0 aro9Δ::KanMX* [ura$^-$ his$^-$ leu$^-$ met$^-$ G418^R]	YKO *MAT*a Strain Collection (open biosystems)
ST892	CEN.PK102-5B	pCfB22, pCfB997, pCfB998	P_{TEF1}::HsDDC P_{PGK1}::BtAANAT-A55P loxP-SpHIS5 P_{TEF1}::GgASMT loxP-KlLEU2 loxP-KlURA3	This study
SCE-iL3-HM-11	CEN.PK102-5B	pCfB1249, pCfB1251, pCfB2529	P_{TEF1}::LrPCBD1 P_{PGK1}::HsDHPR loxP-SpHIS5 P_{TEF1}::RnPTS P_{PGK1}::RnSPR loxP-KlLEU2 P_{PGK1}::HsTPH2$_{146-460}$ loxP-KlURA3	This study
SCE-iL3-HM-12	CEN.PK102-5B	pCfB1249, pCfB1251, pCfB2528	P_{TEF1}::LrPCBD1 P_{PGK1}::HsDHPR loxP-SpHIS5 P_{TEF1}::RnPTS P_{PGK1}::RnSPR loxP-KlLEU2 P_{PGK1}::SmTPH loxP-KlURA3	This study
SCE-iL3-HM-13	CEN.PK102-5B	pCfB1248, pCfB1251, pCfB2528	P_{TEF1}::PaPCBD1 P_{PGK1}::RnDHPR loxP-SpHIS5 P_{TEF1}::RnPTS P_{PGK1}::RnSPR loxP-KlLEU2 P_{PGK1}::HsTPH2$_{146-460}$ loxP-KlURA3	This study
SCE-iL3-HM-14	CEN.PK102-5B	pCfB1248, pCfB1251, pCfB2528	P_{TEF1}::LrPCBD1 P_{PGK1}::HsDHPR loxP-SpHIS5 P_{TEF1}::RnPTS P_{PGK1}::RnSPR loxP -KlLEU2 P_{PGK1}::SmTPH loxP-KlURA3	This study
SCE-iL3-HM-19	SCE-iL3-HM-11	SpHIS5, KlLEU2 and KlURA3 markers removed by CreA-loxP recombination	P_{TEF1}::LrPCBD1 P_{PGK1}::HsDHPR P_{TEF1}::RnPTS P_{PGK1}::RnSPR P_{PGK1}::HsTPH2$_{146-460}$ [ura$^-$ his$^-$ leu$^-$]	This study
SCE-iL3-HM-20	SCE-iL3-HM-12	SpHIS5, KlLEU2 and KlURA3 markers removed by CreA-loxP recombination	P_{TEF1}::LrPCBD1 P_{PGK1}::HsDHPR P_{TEF1}::RnPTS P_{PGK1}::RnSPR P_{PGK1}::SmTPH [ura$^-$ his$^-$ leu$^-$]	This study
SCE-iL3-HM-21	SCE-iL3-HM-13	SpHIS5, KlLEU2 and KlURA3 markers removed by CreA-loxP recombination	P_{TEF1}::PaPCBD1 P_{PGK1}::RnDHPR P_{TEF1}::RnPTS P_{PGK1}::RnSPR P_{PGK1}::HsTPH2$_{146-460}$ [ura$^-$ his$^-$ leu$^-$]	This study
SCE-iL3-HM-22	SCE-iL3-HM-14	SpHIS5, KlLEU2 and KlURA3 markers removed by CreA-loxP recombination	P_{TEF1}::LrPCBD1 P_{PGK1}::HsDHPR P_{TEF1}::RnPTS P_{PGK1}::RnSPR P_{PGK1}::SmTPH [ura$^-$ his$^-$ leu$^-$]	This study
SCE-iL3-HM-23	SCE-iL3-HM-19	pCfB1252, pCfB2628	P_{TEF1}::LrPCBD1 P_{PGK1}::HsDHPR P_{TEF1}::RnPTS P_{PGK1}::RnSPR P_{PGK1}::HsTPH2$_{146-460}$ P_{TEF1}::HsDDC P_{PGK1}::BtAANAT loxP-SpHIS5 P_{TEF1}::HsASMT loxP-KlLEU2 [ura$^-$]	This study
SCE-iL3-HM-24	SCE-iL3-HM-20	pCfB1252, pCfB2628	P_{TEF1}::LrPCBD1 P_{PGK1}::HsDHPR P_{TEF1}::RnPTS P_{PGK1}::RnSPR P_{PGK1}::SmTPH P_{TEF1}::HsDDC P_{PGK1}::BtAANAT loxP-SpHIS5 P_{TEF1}::HsASMT loxP-KlLEU2 [ura$^-$]	This study

Table s4 continued

**Strain name**	**Parent strain (chassis)**	**Added plasmid**	**Relevant genotype**	**Reference/ Source**
SCE-iL3-HM-25	SCE-iL3-HM-21	pCfB1252, pCfB2628	P_{TEF1}::PaPCBD1 P_{PGK1}::RnDHPR<br>P_{TEF1}::RnPTS P_{PGK1}::RnSPR<br>P_{PGK1}::HsTPH2$_{146-460}$<br>P_{TEF1}::HsDDC P_{PGK1}::BtAANAT loxP-SpHIS5<br>P_{TEF1}::HsASMT loxP-KlLEU2 $[ura^-]$	This study
SCE-iL3-HM-35	SCE-iL3-HM-22	pCfB1252, pCfB2628	P_{TEF1}::LrPCBD1 P_{PGK1}::HsDHPR<br>P_{TEF1}::RnPTS P_{PGK1}::RnSPR P_{PGK1}::SmTPH<br>P_{TEF1}::HsDDC P_{PGK1}::BtAANAT loxP-SpHIS5<br>P_{TEF1}::HsASMT loxP-KlLEU2 $[ura^-]$	This study
SCE-iL3-HM-36	SCE-iL3-HM-23	pCfB255	P_{TEF1}::LrPCBD1 P_{PGK1}::HsDHPR<br>P_{TEF1}::RnPTS P_{PGK1}::RnSPR<br>P_{PGK1}::HsTPH2$_{146-460}$<br>P_{TEF1}::HsDDC P_{PGK1}::BtAANAT loxP-SpHIS5<br>P_{TEF1}::HsASMT loxP-KlLEU2<br>loxP-KlURA3	This study
SCE-iL3-HM-37	SCE-iL3-HM-24	pCfB255	P_{TEF1}::LrPCBD1 P_{PGK1}::HsDHPR<br>P_{TEF1}::RnPTS P_{PGK1}::RnSPR P_{PGK1}::SmTPH<br>P_{TEF1}::HsDDC P_{PGK1}::BtAANAT loxP-SpHIS5<br>P_{TEF1}::HsASMT loxP-KlLEU2<br>loxP-KlURA3	This study
SCE-iL3-HM-38	SCE-iL3-HM-25	pCfB255	P_{TEF1}::PaPCBD1 P_{PGK1}::RnDHPR<br>P_{TEF1}::RnPTS P_{PGK1}::RnSPR<br>P_{PGK1}::HsTPH2$_{146-460}$<br>P_{TEF1}::HsDDC P_{PGK1}::BtAANAT loxP-SpHIS5<br>P_{TEF1}::HsASMT loxP-KlLEU2<br>loxP-KlURA3	This study
SCE-iL3-HM-39	SCE-iL3-HM-35	pCfB255	P_{TEF1}::LrPCBD1 P_{PGK1}::HsDHPR<br>P_{TEF1}::RnPTS P_{PGK1}::RnSPR P_{PGK1}::SmTPH<br>P_{TEF1}::HsDDC P_{PGK1}::BtAANAT loxP-SpHIS5<br>P_{TEF1}::HsASMT loxP-KlLEU2<br>loxP-KlURA3	This study
SCE-iL3-HM-40	SCE-iL3-HM-23	pCfB2773	P_{TEF1}::LrPCBD1 P_{PGK1}::HsDHPR<br>P_{TEF1}::RnPTS P_{PGK1}::RnSPR<br>P_{PGK1}::HsTPH2$_{146-460}$<br>P_{TEF1}::HsDDC P_{PGK1}::BtAANAT loxP-SpHIS5<br>P_{TEF1}::HsASMT loxP-KlLEU2<br>(P_{PGK1}::HsTPH$_{146-460}$ loxP-KlURA3-degron)$_n$ [a)]	This study
SCE-iL3-HM-41	SCE-iL3-HM-24	pCfB2772	P_{TEF1}::LrPCBD1 P_{PGK1}::HsDHPR<br>P_{TEF1}::RnPTS P_{PGK1}::RnSPR P_{PGK1}::SmTPH<br>P_{TEF1}::HsDDC P_{PGK1}::BtAANAT loxP-SpHIS5<br>P_{TEF1}::HsASMT loxP-KlLEU2<br>(P_{PGK1}::SmTPH loxP-KlURA3-degron)$_n$ [a)]	This study

Table s4 continued				
**Strain name**	**Parent strain (chassis)**	**Added plasmid**	**Relevant genotype***	**Reference/ Source**
SCE-iL3-HM-42	SCE-iL3-HM-25	pCfB2773	P_{TEF1}::PaPCBD1 P_{PGK1}::RnDHPR<br>P_{TEF1}::RnPTS P_{PGK1}::RnSPR<br>P_{PGK1}::HsTPH2$_{146-460}$<br>P_{TEF1}::HsDDC P_{PGK1}::BtAANAT loxP-SpHIS5<br>P_{TEF1}::HsASMT loxP-KlLEU2<br>(P_{PGK1}::HsTPH$_{146-460}$ loxP-KlURA3-degron)$_n$ [a)]	This study
SCE-iL3-HM-43	SCE-iL3-HM-35	pCfB2772	P_{TEF1}::LrPCBD1 P_{PGK1}::HsDHPR<br>P_{TEF1}::RnPTS P_{PGK1}::RnSPR P_{PGK1}::SmTPH<br>P_{TEF1}::HsDDC P_{PGK1}::BtAANAT loxP-SpHIS5<br>P_{TEF1}::HsASMT loxP-KlLEU2<br>(P_{PGK1}::SmTPH loxP-KlURA3-degron)$_n$ [a)]	This study
SCE-iL3-HM-44	SCE-iL3-HM-43 clone 7	pCfB3337	P_{TEF1}::LrPCBD1 P_{PGK1}::HsDHPR<br>P_{TEF1}::RnPTS P_{PGK1}::RnSPR P_{PGK1}::SmTPH<br>P_{TEF1}::HsDDC P_{PGK1}::BtAANAT loxP-SpHIS5<br>P_{TEF1}::HsASMT loxP-KlLEU2<br>(P_{PGK1}::SmTPH loxP-KlURA3-degron)$_n$ [a)]<br>P_{TEF1}::HsASMT NatMX (2µ) [b)] [ClonNat^R]	This study
SCE-iL3-HM-45	SCE-iL3-HM-43 clone 17	pCfB3337	P_{TEF1}::LrPCBD1 P_{PGK1}::HsDHPR<br>P_{TEF1}::RnPTS P_{PGK1}::RnSPR P_{PGK1}::SmTPH<br>P_{TEF1}::HsDDC P_{PGK1}::BtAANAT loxP-SpHIS5<br>P_{TEF1}::HsASMT loxP-KlLEU2<br>(P_{PGK1}::SmTPH loxP-KlURA3-degron)$_n$ [a)]<br>P_{TEF1}::HsASMT NatMX (2µ) [b)] [ClonNat^R]	This study
SCE-iL3-HM-47	CEN.PK 102-5B	pCfB2628, pCfB1252	P_{TEF1}::HsDDC P_{PGK1}::BtAANAT loxP-SpHIS5<br>P_{TEF1}::HsASMT loxP-KlLEU2	This study
SCE-iL3-HM-48	SCE-iL3-HM-47	pCfB2529	P_{TEF1}::HsDDC P_{PGK1}::BtAANAT loxP-SpHIS5<br>P_{TEF1}::HsASMT loxP-KlLEU2<br>P_{PGK1}::HsTPH2$_{146-460}$ loxP-KlURA3	This study
SCE-iL3-HM-49	SCE-iL3-HM-47	pCfB2528	P_{TEF1}::HsDDC P_{PGK1}::BtAANAT loxP-SpHIS5<br>P_{TEF1}::HsASMT loxP-KlLEU2<br>P_{PGK1}::SmTPH loxP-KlURA3	This study
SCE-iL3-HM-50	SCE-iL3-HM-47	pCfB2773	P_{TEF1}::HsDDC P_{PGK1}::BtAANAT loxP-SpHIS5<br>P_{TEF1}::HsASMT loxP-KlLEU2<br>(P_{PGK1}::HsTPH$_{146-460}$ loxP-KlURA3-degron)$_n$ [a)]	This study
SCE-iL3-HM-51	SCE-iL3-HM-47	pCfB2772	P_{TEF1}::HsDDC P_{PGK1}::BtAANAT loxP-SpHIS5<br>P_{TEF1}::HsASMT loxP-KlLEU2<br>(P_{PGK1}::SmTPH loxP-KlURA3-degron)$_n$ [a)]	This study
SCE-iL3-HM-54	SCE-iL3-HM-43 clone 61	*aro9*Δ::KanMX deletion cassette	P_{TEF1}::LrPCBD1 P_{PGK1}::HsDHPR<br>P_{TEF1}::RnPTS P_{PGK1}::RnSPR P_{PGK1}::SmTPH<br>P_{TEF1}::HsDDC P_{PGK1}::BtAANAT loxP-SpHIS5<br>P_{TEF1}::HsASMT loxP-KlLEU2<br>(P_{PGK1}::SmTPH loxP-KlURA3-degron)$_n$ [a)]<br>*aro9*Δ::KanMX [G418^R]	This study

Table s4 continued				
**Strain name**	**Parent strain (chassis)**	**Added plasmid**	**Relevant genotype***	**Reference/ Source**
SCE-iL3-HM-57	SCE-iL3-HM-43 clone 61	pCfB4149	P_{TEF1}::LrPCBD1 P_{PGK1}::HsDHPR<br>P_{TEF1}::RnPTS P_{PGK1}::RnSPR P_{PGK1}::SmTPH<br>P_{TEF1}::HsDDC P_{PGK1}::BtAANAT loxP-SpHIS5<br>P_{TEF1}::HsASMT loxP-KlLEU2<br>(P_{PGK1}::SmTPH loxP-KlURA3-degron)$_n$ [a)]<br>P_{TDH3}::SeACS loxP-KanMX [G418^R]	This study
SCE-iL3-HM-60	SCE-iL3-HM-43 clone 61	pCfB4150	P_{TEF1}::LrPCBD1 P_{PGK1}::HsDHPR<br>P_{TEF1}::RnPTS P_{PGK1}::RnSPR P_{PGK1}::SmTPH<br>P_{TEF1}::HsDDC P_{PGK1}::BtAANAT loxP-SpHIS5<br>P_{TEF1}::HsASMT loxP-KlLEU2<br>(P_{PGK1}::SmTPH loxP-KlURA3-degron)$_n$ [a)]<br>P_{TDH3}::ScALD6 loxP-KanMX [G418^R]	This study
SCE-iL3-HM-63	SCE-iL3-HM-43 clone 61	pCfB4151	P_{TEF1}::LrPCBD1 P_{PGK1}::HsDHPR<br>P_{TEF1}::RnPTS P_{PGK1}::RnSPR P_{PGK1}::SmTPH<br>P_{TEF1}::HsDDC P_{PGK1}::BtAANAT loxP-SpHIS5<br>P_{TEF1}::HsASMT loxP-KlLEU2<br>(P_{PGK1}::SmTPH loxP-KlURA3-degron)$_n$ [a)]<br>P_{TDH3}::ScSAM2 loxP-KanMX [G418^R]	This study
SCE-iL3-HM-66	SCE-iL3-HM-43 clone 61	pCfB4152	P_{TEF1}::LrPCBD1 P_{PGK1}::HsDHPR<br>P_{TEF1}::RnPTS P_{PGK1}::RnSPR P_{PGK1}::SmTPH<br>P_{TEF1}::HsDDC P_{PGK1}::BtAANAT loxP-SpHIS5<br>P_{TEF1}::HsASMT loxP-KlLEU2<br>(P_{PGK1}::SmTPH loxP-KlURA3-degron)$_n$ [a)]<br>P_{TDH3}::ScERC1 loxP-KanMX [G418^R]	This study

a) (DNA fragment)$_n$ denotes multi-copy integration of the DNA fragment into TY2 regions of the genome.

b) (2μ) refers to an episomal high-copy plasmid with 2μ origin.

Table s5: Production titers of melatonin cell factories. Strains carry genes for overexpressing RnPTS, RnSPR, HsDDC, BtAANAT, HsASMT, and two variants of TPH, PCBD1, and DHPR, respectively. Cells were cultured in mineral medium, the supernatant extracted with ethanol, and metabolites measured by LC-ESI-MS. Standard deviations were calculated based on three individual clones (CEN.PK113-7D, SCE-iL3-HM-36 to -42) or six individual clones (SCE-iL3-HM-43).

Melatonin production from glucose - Single TPH intergration

Strain ID	5-HTP (mg L^{-1})	serotonin (mg L^{-1})	*N*-acetylserotonin (mg L^{-1})	melatonin (mg L^{-1})	BH4 recycling genes	TPH genes
CEN.PK113-7D	0.00 ± 0.00	0.00 ± 0.00	0.00 ± 0.00	0.00 ± 0.00	-	-
SCE-iL3-HM-36	0.00 ± 0.00	0.11 ± 0.19	3.99 ± 0.70	0.41 ± 0.16	LrPCBD1/HsDHPR	HsTPH
SCE-iL3-HM-38	0.00 ± 0.00	2.43 ± 4.21	7.47 ± 0.91	0.76 ± 0.20	PaPCBD1/RnDHPR	HsTPH
SCE-iL3-HM-37	0.00 ± 0.00	0.00 ± 0.00	4.09 ± 0.83	0.27 ± 0.16	LrPCBD1/HsDHPR	SmTPH
SCE-iL3-HM-39	0.00 ± 0.00	0.00 ± 0.00	9.14 ± 1.90	0.82 ± 0.19	PaPCBD1/RnDHPR	SmTPH

Melatonin production from glucose - Multiple TPH intergration

Strain ID	5-HTP (mg L^{-1})	serotonin (mg L^{-1})	*N*-acetylserotonin (mg L^{-1})	melatonin (mg L^{-1})	BH4 recycling genes	TPH genes
SCE-iL3-HM-40	0.00 ± 0.00	0.17 ± 0.30	4.88 ± 1.70	0.40 ± 0.07	LrPCBD1/HsDHPR	HsTPH
SCE-iL3-HM-42	0.09 ± 0.02	4.09 ± 0.76	7.24 ± 0.85	0.90 ± 0.21	PaPCBD1/RnDHPR	HsTPH
SCE-iL3-HM-41	0.00 ± 0.00	0.13 ± 0.03	1.47 ± 0.37	0.03 ± 0.00	LrPCBD1/HsDHPR	SmTPH
SCE-iL3-HM-43	0.22 ± 0.08	9.53 ± 3.57	16.69 ± 0.60	1.93 ± 0.19	PaPCBD1/RnDHPR	SmTPH

Table s6: SmTPH and HsTPH$_{146-460}$ copy number and mRNA levels in the respective production strains. Strains were cultured in SC-ura dropout medium and harvested for DNA or RNA extraction, and subsequently QPCR or RT-QPCR was performed with primer pairs for SmTPH, HsTPH$_{146-460}$, and Sc*ACT1* as reference. Relative SmTPH and HsTPH$_{146-460}$ copy number and mRNA levels were found by normalizing to Sc*ACT1*. All reactions were performed in triplicate and the standard deviations calculated.

TPH copy number and mRNA expression levels normalized to ACT1

Strain ID	SmTPH copy number	Relative SmTPH mRNA	Clonal variation of mRNA expression
SCE-iL3-HM-37_9	1.07 ± 0.09	0.61 ± 0.14	-
SCE-iL3-HM-39_4	0.98 ± 0.13	2.12 ± 0.09	-
SCE-iL3-HM-41_4	7.19 ± 0.28	11.69 ± 0.20	SCE-iL3-HM-41
SCE-iL3-HM-41_6	7.37 ± 0.18	34.42 ± 2.86	23.06 ± 16.07
SCE-iL3-HM-43_7	2.34 ± 0.09	13.51 ± 1.35	SCE-iL3-HM-43
SCE-iL3-HM-43_17	11.18 ± 0.83	15.35 ± 0.98	13.68 ± 1.59
SCE-iL3-HM-43_61	2.72 ± 0.84	12.18 ± 1.35	
CEN.PK113-7D	0.00 ± 0.00	0.00 ± 0.00	-
SCE-iL3-HM-49_1	0.75 ± 0.03	0.75 ± 0.05	-
SCE-iL3-HM-51_1	12.66 ± 0.72	53.07 ± 7.64	SCE-iL3-HM-51
SCE-iL3-HM-51_2	1.48 ± 0.07	17.94 ± 0.76	35.51 ± 24.85

Strain ID	HsTPH copy number	Relative HsTPH mRNA	Clonal variation of mRNA expression
SCE-iL3-HM-36_4	1.09 ± 0.08	0.98 ± 0.04	-
SCE-iL3-HM-38_6	0.97 ± 0.04	1.40 ± 0.09	-
SCE-iL3-HM-40_3	10.14 ± 0.49	14.32 ± 1.48	SCE-iL3-HM-40
SCE-iL3-HM-40_18	7.27 ± 0.20	18.74 ± 3.51	16.53 ± 3.13
SCE-iL3-HM-42_23	7.96 ± 0.43	10.43 ± 1.86	SCE-iL3-HM-42
SCE-iL3-HM-42_27	9.09 ± 1.69	10.32 ± 0.58	10.38 ± 0.08
CEN.PK113-7D	0.00 ± 0.00	0.00 ± 0.00	-
SCE-iL3-HM-48_1	0.76 ± 0.04	0.91 ± 0.08	-
SCE-iL3-HM-50_1	10.17 ± 0.19	22.13 ± 2.17	SCE-iL3-HM-50
SCE-iL3-HM-50_2	8.27 ± 1.00	24.36 ± 5.29	23.24 ± 1.58

Table s7: Production titers of the best producing melatonin strain in different cultivation media. Analysis of the non-producing strain CEN.PK 113-7D and the best producing strain SCE-iL3-HM-43 that carries genes for overexpressing RnPTS, RnSPR, PaPCBD1, RnDHPR, HsDDC, BtAANAT, HsASMT, and SmTPH(Ty2) under different cultivation conditions. Strains were cultivated in mineral medium or in FIT medium, the total cell suspension extracted with ethanol, and metabolites measured by LC-ESI-MS. Standard deviations were calculated based on two independent clones.

Melatonin production from glucose - Mineral medium (MM) vs FIT medium

Strain ID	media	serotonin (mg/l)	*N*-acetylserotonin (mg/l)	melatonin (mg/l)	Fold-change in melatonin production*	*p*-value*
CEN.PK113-7D	MM	0.00 ± 0.00	0.00 ± 0.00	0.00 ± 0.00	-	-
SCE-iL3-HM-43		65.91 ± 0.99	22.07 ± 0.20	1.42 ± 0.30	-	-
CEN.PK113-7D	FIT	0.00 ± 0.00	0.00 ± 0.00	0.00 ± 0.00	-	NA
SCE-iL3-HM-43		2.02 ± 0.01	43.27 ± 7.05	13.15 ± 2.05	9.23	0.0016, **

* fold-change and *p*-values were calculated compared to the identical strain in mineral medium.
** *p*-value highly significant

Table s8: Production titers of melatonin producing strains with additional modifications. All strains are offspring of SCE-iL3-HM-43 clone 61, and carry genes for overexpressing RnPTS, RnSPR, PaPCBD1, RnDHPR, HsDDC, BtAANAT, HsASMT, and SmTPH(Ty2). In addition, either Sc*ARO9* is deleted, or one of the following genes is overexpressed: SeACS, Sc*ALD6*, Sc*SAM2*, or Sc*ERC1*. Cells were cultured in mineral medium or FIT medium, total cell suspension extracted with ethanol, and metabolites measured by LC-ESI-MS. Standard deviations are based on biological triplicates.

Melatonin production from glucose with additional metabolic engineering - Mineral medium

Strain ID	additional genetic modification	serotonin (mg/l)	*N*-acetylserotonin (mg/l)	melatonin (mg/l)	Fold-change in melatonin production*	*p*-value*
CEN.PK113-7D	-	0.00 ± 0.00	0.00 ± 0.00	0.00 ± 0.00	-	-
SCE-iL3-HM-43	-	65.91 ± 0.99	22.07 ± 0.20	1.42 ± 0.30	-	-
SCE-iL3-HM-54	*aro9*Δ	56.56 ± 15.06	22.26 ± 4.21	1.65 ± 0.29	1.16	0.4654, ns
SCE-iL3-HM-57	SeACS	64.32 ± 1.41	22.01 ± 0.09	1.73 ± 0.05	1.21	0.1600, ns
SCE-iL3-HM-60	Sc*ALD6*	30.04 ± 0.35	21.78 ± 0.53	1.52 ± 0.07	1.07	0.5992, ns
SCE-iL3-HM-63	Sc*SAM2*	64.71 ± 1.87	20.08 ± 0.81	1.44 ± 0.10	1.01	0.9857, ns
SCE-iL3-HM-66	Sc*ERC1*	51.35 ± 11.59	20.68 ± 2.35	1.57 ± 0.17	1.11	0.5362, ns

Melatonin production from glucose with additional metabolic engineering - FIT medium

Strain ID	additional genetic modification	serotonin (mg/l)	*N*-acetylserotonin (mg/l)	melatonin (mg/l)	Fold-change in melatonin production*	*p*-value*
CEN.PK113-7D	-	0.00 ± 0.00	0.00 ± 0.00	0.00 ± 0.00	-	-
SCE-iL3-HM-43	-	2.02 ± 0.01	43.27 ± 7.05	13.15 ± 2.05	-	-
SCE-iL3-HM-54	*aro9*Δ	2.64 ± 0.27	43.40 ± 0.66	13.14 ± 0.53	1.00	0.9915, ns
SCE-iL3-HM-57	SeACS	1.90 ± 0.33	43.04 ± 3.30	13.84 ± 2.09	1.05	0.7400, ns
SCE-iL3-HM-60	Sc*ALD6*	2.61 ± 0.07	44.77 ± 2.08	14.50 ± 0.57	1.10	0.3291, ns
SCE-iL3-HM-63	Sc*SAM2*	2.56 ± 0.14	42.83 ± 3.43	10.93 ± 0.19	0.83	0.1344, ns
SCE-iL3-HM-66	Sc*ERC1*	2.53 ± 0.68	33.28 ± 10.82	10.72 ± 1.90	0.82	0.2664, ns

* fold-change and *p*-value were calculated compared to the parent strain (SCE-iL3-HM-43) in the respective medium. ns = not significant.

Sequence Listing. All sequences are protected by the filed patent application [7].

SEQ ID NO: 1

BtAANAT->

1 ATGAGCACCC CGAGCATTCA TTGTCTGAAA CCGAGTCCGC TGCATCTGCC GAGCGGTATT

61 CCGGGTAGTC CGGGTCGTCA GCGTCGCCAT ACCCTGCCTG CAAATGAATT TCGTTGTCTG

121 ACACCGGAAG ATGCAGCCGG TGTTTTTGAA ATTGAACGTG AAGCATTTAT TAGCGTGAGC

181 GGTAATTGTC CGCTGAACCT GGATGAAGTT CGTCATTTTC TGACCCTGTG TCCGGAACTG

241 AGCCTGGGTT GGTTTGTTGA AGGTCGTCTG GTTGCATTTA TCATTGGTAG CCTGTGGGAT

301 GAAGAACGTC TGACCCAAGA AAGCCTGACC CTGCATCGTC CGGGTGGTCG TACCGCACAT

361 CTGCATGCAC TGGCAGTTCA TCATTCATTT CGTCAGCAGG GCAAAGGTAG CGTTCTGCTG

421 TGGCGTTATC TGCAACATGC CGGTGGTCAG CCTGCCGTTC GTCGTGCAGT TCTGATGTGT

481 GAAGATGCAC TGGTTCCGTT TTATCAGCGT TTTGGTTTTC ATCCGGCAGG TCCGTGTGCA

541 GTTGTTGTGG GTAGCCTGAC CTTTACCGAA ATGCATTGTA GCCTGCGTGG TCATGCAGCA

601 CTGCGTCGTA ATAGCGATCG TTAA

SEQ ID NO: 2

HsASMT<-

1 ATGGGTAGCA GCGAAGATCA GGCATATCGT CTGCTGAATG ATTATGCCAA TGGTTTTATG

61 GTTAGCCAGG TTCTGTTTGC AGCATGTGAA CTGGGTGTTT TTGATCTGCT GGCAGAAGCA

121 CCGGGTCCGC TGGATGTTGC AGCAGTTGCA GCCGGTGTTC GTGCAAGCGC ACATGGCACC

181 GAACTGCTGC TGGATATTTG TGTTAGCCTG AAACTGCTGA AAGTTGAAAC CCGTGGTGGT

241 AAAGCATTTT ATCGTAATAC CGAACTGAGC AGCGATTATC TGACCACCGT TAGCCCGACC

301 AGCCAGTGTA GCATGCTGAA ATATATGGGT CGTACCAGCT ATCGTTGTTG GGGTCATCTG

361 GCAGATGCAG TTCGTGAAGG TCGTAATCAG TATCTGGAAA CCTTTGGTGT TCCGGCAGAA

421 GAACTGTTTA CCGCAATTTA TCGTAGCGAA GGTGAACGTC TGCAGTTTAT GCAGGCACTG

481 CAAGAGGTTT GGAGCGTTAA TGGTCGTAGC GTTCTGACCG CATTTGATCT GAGCGTTTTT

541 CCGCTGATGT GTGATCTGGG TGGTGGTGCC GGTGCACTGG CAAAAGAATG TATGAGCCTG

601 TATCCGGGTT GTAAAATTAC CGTTTTTGAT ATTCCGGAAG TTGTGTGGAC CGCAAAACAG

661 CATTTTAGCT TTCAAGAAGA GGAACAAATC GACTTTCAAG AGGGCGATTT TTTCAAAGAT

721 CCGCTGCCGG AAGCAGATCT GTATATTCTG GCACGTGTTC TGCATGATTG GGCAGATGGT

781 AAATGCAGCC ATCTGCTGGA ACGTATTTAT CATACCTGTA AACCGGGTGG TGGCATTCTG

841 GTTATTGAAA GCCTGCTGGA TGAAGATCGT CGTGGTCCGC TGCTGACCCA GCTGTATAGC

901 CTGAATATGC TGGTTCAGAC CGAAGGTCAA GAACGTACCC CGACCCATTA TCATATGCTG

961 CTGAGCAGTG CAGGTTTTCG TGATTTTCAG TTCAAAAAAA CCGGTGCCAT TTATGATGCA

1021 ATCCTGGCAC GTAAATAA

SEQ ID NO: 3

HsDDC<-

1 ATGAATGCAA GCGAATTTCG TCGTCGTGGT AAAGAAATGG TTGATTATAT GGCCAACTAC

61 ATGGAAGGTA TTGAAGGTCG TCAGGTTTAT CCGGATGTTG AACCGGGTTA TCTGCGTCCG

121 CTGATTCCGG CAGCAGCACC GCAAGAACCG GATACCTTTG AAGATATTAT CAACGATGTG

181 GAAAAAATCA TCATGCCTGG TGTTACCCAT TGGCATAGCC CGTATTTCTT TGCATATTTT

241 CCGACCGCAA GCAGCTATCC GGCAATGCTG GCAGATATGC TGTGTGGTGC AATTGGTTGT

301 ATTGGTTTTA GCTGGGCAGC AAGTCCGGCA TGTACCGAAC TGGAAACCGT TATGATGGAT

361 TGGCTGGGTA AAATGCTGGA ACTGCCGAAA GCATTTCTGA ATGAAAAAGC CGGTGAAGGT

421 GGTGGTGTTA TTCAGGGTAG CGCAAGCGAA GCAACCCTGG TTGCACTGCT GGCAGCACGT

481 ACCAAAGTTA TTCATCGTCT GCAAGCAGCA TCACCGGAAC TGACCCAGGC AGCAATTATG

541 GAAAAACTGG TTGCATATAG CAGCGATCAG GCACATAGCA GCGTTGAACG TGCAGGTCTG

601 ATTGGTGGTG TGAAACTGAA AGCAATTCCG AGTGATGGTA ATTTTGCAAT GCGTGCAAGC

661 GCACTGCAAG AAGCACTGGA ACGCGATAAA GCAGCCGGTC TGATTCCGTT TTTTATGGTT

721 GCCACCCTGG GTACAACCAC CTGTTGTAGC TTTGATAATC TGCTGGAAGT TGGTCCGATT

781 TGCAACAAAG AAGATATTTG GCTGCACGTT GATGCAGCAT ATGCAGGTAG CGCATTTATT

841 TGTCCGGAAT TTCGCCATCT GCTGAATGGT GTTGAATTTG CAGATAGCTT TAACTTTAAC

901 CCGCATAAAT GGCTGCTGGT GAATTTTGAT TGTAGCGCAA TGTGGGTTAA AAAACGTACC

961 GATCTGACCG GTGCATTTCG TCTGGACCCG ACCTATCTGA AACATAGCCA TCAGGATAGT

1021 GGTCTGATTA CCGATTATCG TCATTGGCAG ATTCCGCTGG GTCGTCGTTT TCGTAGCCTG

1081 AAAATGTGGT TTGTGTTTCG TATGTATGGT GTCAAAGGCC TGCAAGCCTA TATTCGTAAA

1141 CATGTTCAGC TGAGCCATGA ATTTGAAAGC CTGGTTCGTC AAGATCCGCG TTTTGAAATT

1201 TGCGTTGAAG TTATTCTGGG TCTGGTTTGC TTTCGTCTGA AAGGTAGCAA TAAAGTTAAT

1261 GAAGCACTGC TGCAACGTAT CAACAGCGCC AAAAAAATCC ATCTGGTTCC GTGTCATCTG

1321 CGCGATAAAT TTGTTCTGCG TTTTGCCATT TGTAGCCGTA CCGTTGAAAG TGCCCATGTG

1381 CAGCGTGCAT GGGAACATAT TAAAGAACTG GCAGCAGATG TGCTGCGTGC CGAACGTGAA

1441 TAA

SEQ ID NO: 4

HsDHPR->

1 ATGGCTGCTG CTGCAGCTGC TGGTGAAGCT AGAAGAGTTT TGGTTTATGG TGGTAGAGGT

61 GCTTTGGGTT CTAGATGTGT TCAAGCTTTT AGAGCTAGAA ATTGGTGGGT TGCTTCTGTT

121 GATGTTGTCG AAAATGAAGA AGCCTCCGCT TCTATTATCG TTAAGATGAC TGATTCCTTC

181 ACCGAACAAG CTGATCAAGT TACTGCTGAA GTAGGTAAGT TGTTGGGTGA AGAAAAGGTT

241 GATGCTATTT TGTGTGTTGC TGGTGGTTGG GCTGGTGGTA ATGCTAAATC TAAATCTTTG

301 TTCAAGAACT GCGACTTGAT GTGGAAGCAA TCTATTTGGA CTTCCACCAT CTCTTCTCAT

361 TTGGCTACCA AACATTTGAA AGAAGGTGGT TTGTTGACTT TGGCTGGTGC TAAAGCTGCT

421 TTGGATGGTA CTCCAGGTAT GATTGGTTAT GGTATGGCTA AAGGTGCTGT TCATCAATTG

481 TGTCAATCAT TGGCTGGTAA GAATTCTGGT ATGCCACCAG GTGCTGCTGC TATTGCTGTT

541 TTGCCAGTTA CTTTGGATAC TCCAATGAAC AGAAAGTCTA TGCCAGAAGC TGATTTCTCT

601 TCTTGGACTC CATTGGAATT CTTGGTTGAA ACTTTCCATG ATTGGATCAC CGGTAAGAAT

661 AGACCATCTT CTGGTTCCTT GATTCAAGTT GTTACTACTG AAGGTAGAAC CGAATTGACT

721 CCAGCTTACT TCTAA

SEQ ID NO: 5

RnDHPR->

1 ATGGCTGCTT CTGGTGAAGC TAGAAGAGTT TTGGTTTATG GTGGTAGAGG TGCTTTGGGT

61 TCTAGATGTG TTCAAGCTTT TAGAGCTAGA AATTGGTGGG TTGCCTCTAT CGATGTTGTT

121 GAAAATGAAG AAGCTTCCGC CTCTGTTATC GTTAAGATGA CTGATTCTTT CACCGAACAA

181 GCTGATCAAG TTACTGCTGA AGTAGGTAAG TTGTTGGGTG ATCAAAAGGT TGATGCTATT

241 TTGTGTGTTG CTGGTGGTTG GGCTGGTGGT AATGCTAAAT CTAAATCTTT GTTCAAGAAC

301 TGCGACTTGA TGTGGAAGCA ATCTATTTGG ACTTCCACCA TCTCTTCTCA TTTGGCTACC

361 AAACATTTGA AAGAAGGTGG TTTGTTGACT TTGGCTGGTG CTAAAGCTGC TTTGGATGGT

421 ACTCCAGGTA TGATTGGTTA TGGTATGGCT AAAGGTGCTG TTCATCAATT GTGTCAATCA

481 TTGGCTGGTA AGAATTCTGG TATGCCATCT GGTGCTGCTG CTATTGCTGT TTTGCCAGTT

541 ACTTTGGATA CTCCAATGAA CAGAAAGTCT ATGCCAGAAG CTGATTTCTC TTCTTGGACT

601 CCATTGGAAT TCTTGGTTGA AACTTTCCAT GATTGGATCA CCGGTAACAA AAGACCAAAT

661 TCCGGTTCCT TGATTCAAGT TGTTACTACT GATGGTAAGA CCGAATTGAC TCCAGCTTAC

721 TTCTAA

SEQ ID NO: 6

LrPCBD1<-

```
1       ATGGTCAAGT TGTTCCCATC TGAAAATGCT AGAAGATGGC ATAGATGGAA CCACGAAGTT
61      TTGTTGTTGG TCAACATCCA ATGCTCATTG AAACAACCAT TGTGGTCTGC TGAAGGTAAG
121     GTTGATAAGA ACAGAGAAAA GTGTGCTGCC TTCGTTTACA GATTGGTTGA AATTCAAGAT
181     GCCAGAATTT GA
```

SEQ ID NO: 7

PaPCBD1<-

```
1       ATGACTGCTT TGACTCAAGC TCATTGTGAA GCTTGTAGAG CTGATGCTCC ACATGTTTCT
61      GATGAAGAAT TGCCAGTTTT GTTGAGACAA ATCCCAGATT GGAACATCGA AGTTAGAGAT
121     GGTATCATGC AATTGGAAAA GGTCTACTTG TTCAAGAACT TCAAACATGC TTTGGCTTTC
181     ACTAACGCTG TTGGTGAAAT TTCTGAAGCT GAAGGTCATC ATCCAGGTTT GTTGACTGAA
241     TGGGGTAAAG TTACTGTTAC TTGGTGGTCC CATTCTATCA AAGGTTTACA CAGAAACGAT
301     TTCATTATGG CTGCTAGAAC TGATGAAGTT GCTAAAACTG CTGAAGGTAG AAAGTAA
```

SEQ ID NO: 8

RnPTS<-

```
1       ATGAACGCGG CGGTTGGCCT TCGGCGCCGC GCGCGATTGT CGCGCCTCGT GTCCTTCAGC
61      GCGAGCCACC GGCTGCACAG CCCATCTCTG AGTGCTGAGG AGAACTTGAA AGTGTTTGGG
121     AAATGCAACA ATCCGAATGG CCATGGGCAC AACTATAAAG TTGTGGTGAC AATTCATGGA
181     GAGATCGATC CGGTTACAGG AATGGTTATG AATTTGACTG ACCTCAAAGA ATACATGGAG
241     GAGGCCATTA TGAAGCCCCT TGATCACAAG AACCTGGATC TGGATGTGCC ATACTTTGCA
301     GATGTTGTAA GCACGACAGA AAATGTAGCT GTCTATATCT GGGAGAACCT GCAGAGACTT
361     CTTCCAGTGG GAGCTCTCTA TAAAGTAAAA GTGTATGAAA CTGACAACAA CATTGTGGTC
421     TACAAAGGAG AATAA
```

SEQ ID NO: 9

RnSPR->

```
1       ATGGAAGGAG GCAGGCTAGG TTGCGCTGTC TGCGTGCTGA CCGGGGCTTC CCGGGGCTTC
61      GGCCGCGCCC TGGCCCCGCA GCTGGCCGGG TTGCTGTCGC CCGGTTCGGT GTTGCTTCTA
```

121 AGCGCACGCA GTGACTCGAT GCTGCGGCAA CTGAAGGAGG AGCTCTGTAC GCAGCAGCCG

181 GGCCTGCAAG TGGTGCTGGC AGCCGCCGAT TTGGGCACCG AGTCCGGCGT GCAACAGTTG

241 CTGAGCGCGG TGCGCGAGCT CCCTAGGCCC GAGAGGCTGC AGCGCCTCCT GCTCATCAAC

301 AATGCAGGCA CTCTTGGGGA TGTTTCCAAA GGCTTCCTGA ACATCAATGA CCTAGCTGAG

361 GTGAACAACT ACTGGGCCCT GAACCTAACC TCCATGCTCT GCTTGACCAC CGGCACCTTG

421 AATGCCTTCT CCAATAGCCC TGGCCTGAGC AAGACTGTAG TTAACATCTC ATCTCTGTGT

481 GCCCTGCAGC CCTTCAAGGG CTGGGGACTC TACTGTGCAG GGAAGGCTGC CCGAGACATG

541 TTATACCAGG TCCTGGCTGT TGAGGAACCC AGTGTGAGGG TGCTGAGCTA TGCCCCAGGT

601 CCCCTGGACA CCAACATGCA GCAGTTGGCC CGGGAAACCT CCATGGACCC AGAGTTGAGG

661 AGCAGACTGC AGAAGTTGAA TTCTGAGGGG GAGCTGGTGG ACTGTGGGAC TTCAGCCCAG

721 AAACTGCTGA GCTTGCTGCA AAGGGACACC TTCCAATCTG GAGCCCACGT GGACTTCTAT

781 GACATTTAA

SEQ ID NO: 10

HsTPH2$_{146-460}$ ->

1 ATGGAACTGG AAGATGTTCC GTGGTTTCCG CGTAAAATTA GCGAACTGGA TAAATGTAGC

61 CATCGTGTTC TGATGTATGG TAGTGAACTG GATGCAGATC ATCCGGGTTT TAAAGATAAT

121 GTTTATCGTC AGCGTCGCAA ATATTTCGTT GATGTTGCAA TGGGTTACAA ATATGGTCAG

181 CCGATTCCGC GTGTTGAATA TACCGAAGAA GAAACCAAAA CCTGGGGTGT TGTTTTTCGT

241 GAACTGAGCA AACTGTATCC GACCCATGCA TGTCGTGAAT ATCTGAAAAA CTTTCCGCTG

301 CTGACCAAAT ATTGTGGTTA TCGTGAAGAT AACGTTCCGC AGCTGGAAGA TGTGAGCATG

361 TTTCTGAAAG AACGTAGCGG TTTTACCGTT CGTCCGGTTG CAGGTTATCT GAGTCCGCGT

421 GATTTTCTGG CAGGTCTGGC ATATCGTGTT TTTCATTGTA CCCAGTATAT TCGCCATGGT

481 AGCGATCCGC TGTATACTCC GGAACCGGAT ACCTGTCATG AACTGCTGGG TCATGTGCCG

541 CTGCTGGCAG ATCCGAAATT TGCACAGTTT AGCCAAGAAA TTGGTCTGGC AAGCCTGGGT

601 GCAAGTGATG AAGATGTGCA GAAACTGGCA ACCTGTTATT TCTTTACCAT TGAATTTGGC

661 CTGTGCAAAC AAGAGGGTCA GCTGCGTGCA TATGGTGCAG GTCTGCTGAG CAGCATTGGT

721 GAACTGAAAC ATGCACTGAG CGATAAAGCA TGTGTTAAAG CATTTGATCC GAAAACCACC

781 TGTCTGCAAG AATGTCTGAT TACCACCTTT CAAGAAGCCT ATTTCGTTAG CGAAAGCTTT

841 GAAGAGGCCA AAGAAAAAAT GCGCGATTTT GCCAAAAGCA TTACCCGTCC GTTTAGCGTT

901 TATTTCAATC CGTATACCCA GAGCATCGAG ATCCTGAAAG ATACCTAA

SEQ ID NO: 11

SmTPH->

```
1       ATGATTAGCA CCGAAAGCGA TCTGCGTCGT CAGCTGGATG AAAATGTTCG TAGCGAAGCA
61      GATGAAAGCA CCAAAGAAGA ATGTCCGTAT ATTAACGCAG TTCAGAGCCA TCATCAGAAC
121     GTTCAAGAAA TGAGCATTAT TATCAGCCTG GTGAAAAACA TGAACGATAT GAAAAGCATC
181     ATCAGCATCT TTACCGATCG CAACATTAAC ATCCTGCATA TTGAAAGCCG TCTGGGTCGT
241     CTGAATATGA AAAAACATAC CGAAAAAAGC GAATTTGAGC CGCTGGAACT GCTGGTTCAT
301     GTTGAAGTTC CGTGTATTGA AGTTGAACGT CTGCTGGAAG AACTGAAAAG CTTTAGCAGC
361     TATCGTATTG TTCAGAATCC GCTGATGAAT CTGCCGGAAG CAAAAAATCC GACCCTGGAT
421     GATAAAGTGC CGTGGTTTCC GCGTCATATT TCAGATCTGG ATAAAGTTAG CAATAGCGTG
481     CTGATGTATG GCAAAGAACT GGATGCAGAT CATCCGGGTT TTAAAGATAA AGAATATCGC
541     AAACGTCGCA TGATGTTTGC AGATATTGCA CTGAACTATA AATGGGGTCA GCAGATTCCG
601     ATTGTGGAAT ATACCGAAAT TGAAAAAACC ACCTGGGGTC GTATTTATCG TGAACTGACC
661     CGTCTGTATA AAACCAGCGC ATGTCATGAA TTTCAGAAAA ATCTGGGTCT GCTGCAGGAT
721     AAAGCAGGCT ATAATGAATT TGATCTGCCG CAGCTGCAGG TTGTTAGCGA TTTCCTGAAA
781     GCACGTACCG GTTTTTGTCT GCGTCCGGTT GCAGGTTATC TGAGCGCACG TGATTTTCTG
841     AGCGGTCTGG CATTTCGTGT GTTTTATTGT ACCCAGTATA TTCGTCATCA GGCCGATCCG
901     TTTTATACTC CGGAACCGGA TTGTTGTCAT GAGCTGCTGG GCCATGTTCC GATGCTGGCA
961     GATCCGAAAT TTGCACGTTT TAGCCAAGAA ATTGGTCTGG CAAGCCTGGG CACCAGTGAT
1021    GAAGAAATCA AAAAACTGGC AACCTGCTAC TTCTTTACCA TTGAATTTGG TCTGTGCCGT
1081    CAGGATAATC AGCTGAAAGC ATATGGTGCA GGTCTGCTGA GCAGCGTTGC AGAACTGCAG
1141    CATGCACTGA GCGATAAAGC CGTTATTAAA CCGTTTATTC CGATGAAAGT GATCAACGAA
1201    GAATGCCTGG TTACCACCTT TCAGAATGGT TATTTCGAAA CCAGCAGCTT TGAAGATGCA
1261    ACCCGTCAGA TGCGTGAATT TGTTCGTACC ATTAAACGTC CGTTTGATGT GCATTATAAT
1321    CCGTATACCC AGAGCATCGA AATTATCAAA ACCCCGAAAA GCGTTGCCAA ACTGGTTCAG
1381    GATCTGCAGT TTGAACTGAC AGCAATTAAT GAAAGCCTGC TGAAAATGAA CAAAGAAATT
1441    CGTAGCCAGC AGTTTACCAC CAACAAAATT GTTACCGAAA ATCGCAGCAG CTAA
```

SEQ ID NO: 12

pCfB2772

```
LOCUS       p2772_(pTY2-KlURA3-TAG-PGK1->SmTPH) 7940 bp    DNA     circular
FEATURES             Location/Qualifiers
     terminator      6..176
```

```
                 /label=tCYC1; terminator of S. cerevisiae
     Misc._feature   224..283
                 /label=loxP
     terminator      complement(284..406)
                 /label=tURA3; terminator of K. lactis
     misc_structure  complement(407..457)
                 /label="Degradation tag"
     CDS             complement(458..1258)
                 /label=KlURA3 (minus STOP codon)
     promoter        complement(1259..1757)
                 /label=pURA3; promoter of K. lactis
     Misc._feature   1758..1811
                 /label=loxP
     Misc._feature   1951..2137
                 /label=TY2 3'
     rep_origin      complement(2924..3567)
                 /label=pUC ori
     CDS             complement(3670..4528)
                 /label=AmpR
     Misc._feature   4972..5200
                 /label=TY2 5'
     terminator      complement(5227..5421)
                 /label=tADH1; terminator of S. cerevisiae
     promoter        5440..6423
                 /label=pPGK1; promoter of S. cerevisiae
     CDS             6439..7932
                  /label=SmTPH
ORIGIN
        1 CATTCATCCG CTCTAACCGA AAAGGAAGGA GTTAGACAAC CTGAAGTCTA GGTCCCTATT
       61 TATTTTTTTA TAGTTATGTT AGTATTAAGA ACGTTATTTA TATTTCAAAT TTTTCTTTTT
      121 TTTCTGTACA GACGCGTGTA CGCATGTAAC ATTATACTGA AAACCTTGCT TGAGAAATCG
      181 CGTCAGCTGA AGCTTCGTAC GCTGCAGGTC GACAACCCTT AATGTCGACA ACCCTTAATA
      241 TAACTTCGTA TAATGTATGC TATACGAAGT TATTAGGTCT AGAGATCCCA ATACAACAGA
      301 TCACGTGATC TTTTGTAAGA TGAAGTTGAA GTGAGTGTTG CACCGTGCCA ATGCAGGTGG
```

361 CTATTAGATT AAATATGTGA TTTGTTCTAT TAAGTTTCCT GTATAATTAC AAATGAATAA

421 CGAAATGAGA CAAAGAAGAG AACCAATTTT TACAAGCATG GGGAGCGCTG ATTCTCTTTT

481 GGTACGCTTC CCATCCAGCA TTTCTGTATC TTTCACCTTC AACCTTAGGA TCTCTACCCT

541 TGGCGAAAAG TCCTCTGCCA ACAATGATGA TATCTGATCC ACCACTTACA ACTTCGTCGA

601 CGGTTCTGTA CTGCTGACCC AATGCATCGC CTTTGTCGTC TAAACCTACA CCTGGGGTCA

661 TGATTAGCCA ATCAAACCCT TCTTCTCTTC CTCCCATATC GTTCTGAGCA ATGAACCCAA

721 TAACGAAATC TTTATCACTC TTTGCAATAT CAACGGTACC CTTAGTATAT TCACCGTGTG

781 CTAGAGAACC CTTGGAAGAC AATTCAGCAA GCATCAATAA TCCCCTTGGT TCTTTGGTGA

841 CCTCTTGCGC ACCTTGTTTC AAGCCAGCAA CAATACCAGC ACCAGTAACC CCGTGGGCGT

901 TGGTGATATC AGACCATTCT GCGATACGGT AAACGCCCGA TGTATATTGT AATTTGACTG

961 TGTTACCGAT ATCGGCGAAT TTTCTGTCCT CAAATATCAA GAACTTGTAT TTCTCTGCCA

1021 ATGCTTTCAA TGGAACGACA GTACCCTCAT AACTGAAATC ATCCAAGATA TCAACGTGTG

1081 TTTTCAAAAG GCAAATGTAT GGACCCAACG TTTCAACAAG TTTCAATAGC TCATCAGTCG

1141 AACGAACGTC AAGAGAAGCA CACAAATTGG TCTTCTTTTC ATCCATTAAA CGTAAAAGTT

1201 TCGATGCAAC CGGACTTGCA TGAGTCTCAG CTCTACTGGT ATATGATTTT GTGGACATGG

1261 TGCAACTAAT TGACGGGAGT GTATTGACGC TGGCGTACTG GCTTTCACAA AATGGCCCAA

1321 TCACAACCAC ATCTTAGATA GTTGAAATGA CTTTAGATAA CATCAATTGA GATGAGCTTA

1381 ATCATGTCAA AGCTAAAAGT GTCACCATGA ACGACAATTC TTAAGCAAAT CACGTGATAT

1441 AGATCCACGA ATAACCACCA TTTGATGCTC GAGGCAAGTA ATGTGTGTAA AAAAATGCGT

1501 TACCACCATC CAATGCAGAC CGATCTTCTA CCCAGAATCA CATATATTTA TGTACCGAGT

1561 ACCTTTTTTC TATCTTCCAA TTGCTTCTCC CATATGATTG TCTCCGTAAG CTCGAAATTT

1621 CTAAGTTGGA TTTTAATCTT CACGCAGGAT GACAGTTCGA TGAGCTTCTG AGGAGTGTTT

1681 AGAACATAAT CAGTTTATCC ATGGTCTATC TCTTCTTGTC GCTTTTTCTC CTCGATAGAA

1741 CCTAAATAAA ACGAGCTCTC GAGAACCCTT AATATAACTT CGTATAATGT ATGCTATACG

1801 AAGTTATTAG GTAGGTGATA TCAGATCCAC TAGTGGCCTA TGCACCCAAT TCGCCCTATA

1861 GTGAGTCGTA TTACGCGCGC TCACTGGCCG TCGTTTTACA ACGTCGTGAC TGGGAAAACC

1921 CTGGCGTTAC CCCTGCAGGA CAGACGTCAT TAGTGCTGAG GCATTAATTG ATCAATATAA

1981 AATGATGATA ATAATATTTA TAGAATTGTG TAGAATTGCA GATTCCCTTT TATGGATTCC

2041 TAAATCCTGA GGAGAACTTC TAGTATATTC TACATACCTA ATATTATTGC CTTATTAAAA

2101 ATGGAATCCC AACAATTACA TCAAAATCCA CATTCTCAGA TCTGCGGCCG CACTCAGACC

2161 TGAAGTGAAG TTCCTATACT TTCTAGAGAA TAGGAACTTC TATAGTGAGT CGAATAAGGG

2221 CGACACAAAA TTTATTCTAA ATGCATAATA AATACTGATA ACATCTTATA GTTTGTATTA

2281 TATTTTGTAT TATCGTTGAC ATGTATAATT TTGATATCAA AAACTGATTT TCCCTTTATT

2341 ATTTTCGAGA TTTATTTTCT TAATTCTCTT TAACAAACTA GAAATATTGT ATATACAAAA

2401 AATCATAAAT AATAGATGAA TAGTTTAATT ATAGGTGTTC ATCAATCGAA AAAGCAACGT

2461 ATCTTATTTA AAGTGCGTTG CTTTTTTCTC ATTTATAAGG TTAAATAATT CTCATATATC

2521 AAGCAAAGTG ACAGGCGCCC TTAAATATTC TGACAAATGC TCTTTCCCTA AACTCCCCCC

2581 ATAAAAAAAC CCGCCGAAGC GGGTTTTTAC GTTATTTGCG GATTAACGAT TACTCGTTAT

2641 CAGAACCGCC CAGGGGGCCC GAGCTTAAGA CTGGCCGTCG TTTTACAACA CAGAAAGAGT

2701 TTGTAGAAAC GCAAAAAGGC CATCCGTCAG GGGCCTTCTG CTTAGTTTGA TGCCTGGCAG

2761 TTCCCTACTC TCGCCTTCCG CTTCCTCGCT CACTGACTCG CTGCGCTCGG TCGTTCGGCT

2821 GCGGCGAGCG GTATCAGCTC ACTCAAAGGC GGTAATACGG TTATCCACAG AATCAGGGGA

2881 TAACGCAGGA AAGAACATGT GAGCAAAAGG CCAGCAAAAG GCCAGGAACC GTAAAAAGGC

2941 CGCGTTGCTG GCGTTTTTCC ATAGGCTCCG CCCCCCTGAC GAGCATCACA AAAATCGACG

3001 CTCAAGTCAG AGGTGGCGAA ACCCGACAGG ACTATAAAGA TACCAGGCGT TTCCCCCTGG

3061 AAGCTCCCTC GTGCGCTCTC CTGTTCCGAC CCTGCCGCTT ACCGGATACC TGTCCGCCTT

3121 TCTCCCTTCG GGAAGCGTGG CGCTTTCTCA TAGCTCACGC TGTAGGTATC TCAGTTCGGT

3181 GTAGGTCGTT CGCTCCAAGC TGGGCTGTGT GCACGAACCC CCCGTTCAGC CCGACCGCTG

3241 CGCCTTATCC GGTAACTATC GTCTTGAGTC CAACCCGGTA AGACACGACT TATCGCCACT

3301 GGCAGCAGCC ACTGGTAACA GGATTAGCAG AGCGAGGTAT GTAGGCGGTG CTACAGAGTT

3361 CTTGAAGTGG TGGGCTAACT ACGGCTACAC TAGAAGAACA GTATTTGGTA TCTGCGCTCT

3421 GCTGAAGCCA GTTACCTTCG GAAAAAGAGT TGGTAGCTCT TGATCCGGCA AACAAACCAC

3481 CGCTGGTAGC GGTGGTTTTT TTGTTTGCAA GCAGCAGATT ACGCGCAGAA AAAAAGGATC

3541 TCAAGAAGAT CCTTTGATCT TTTCTACGGG GTCTGACGCT CAGTGGAACG ACGCGCGCGT

3601 AACTCACGTT AAGGGATTTT GGTCATGAGC TTGCGCCGTC CCGTCAAGTC AGCGTAATGC

3661 TCTGCTTTTA CCAATGCTTA ATCAGTGAGG CACCTATCTC AGCGATCTGT CTATTTCGTT

3721 CATCCATAGT TGCCTGACTC CCCGTCGTGT AGATAACTAC GATACGGGAG GGCTTACCAT

3781 CTGGCCCCAG CGCTGCGATG ATACCGCGAG AACCACGCTC ACCGGCTCCG GATTTATCAG

3841 CAATAAACCA GCCAGCCGGA AGGGCCGAGC GCAGAAGTGG TCCTGCAACT TTATCCGCCT

3901 CCATCCAGTC TATTAATTGT TGCCGGGAAG CTAGAGTAAG TAGTTCGCCA GTTAATAGTT

3961 TGCGCAACGT TGTTGCCATC GCTACAGGCA TCGTGGTGTC ACGCTCGTCG TTTGGTATGG

4021 CTTCATTCAG CTCCGGTTCC CAACGATCAA GGCGAGTTAC ATGATCCCCC ATGTTGTGCA

4081 AAAAAGCGGT TAGCTCCTTC GGTCCTCCGA TCGTTGTCAG AAGTAAGTTG GCCGCAGTGT

4141 TATCACTCAT GGTTATGGCA GCACTGCATA ATTCTCTTAC TGTCATGCCA TCCGTAAGAT

4201 GCTTTTCTGT GACTGGTGAG TACTCAACCA AGTCATTCTG AGAATAGTGT ATGCGGCGAC

4261 CGAGTTGCTC TTGCCCGGCG TCAATACGGG ATAATACCGC GCCACATAGC AGAACTTTAA

4321 AAGTGCTCAT CATTGGAAAA CGTTCTTCGG GGCGAAAACT CTCAAGGATC TTACCGCTGT

4381 TGAGATCCAG TTCGATGTAA CCCACTCGTG CACCCAACTG ATCTTCAGCA TCTTTTACTT

4441 TCACCAGCGT TTCTGGGTGA GCAAAAACAG GAAGGCAAAA TGCCGCAAAA AAGGGAATAA

4501 GGGCGACACG GAAATGTTGA ATACTCATAT TCTTCCTTTT TCAATATTAT TGAAGCATTT

4561 ATCAGGGTTA TTGTCTCATG AGCGGATACA TATTTGAATG TATTTAGAAA AATAAACAAA

4621 TAGGGGTCAG TGTTACAACC AATTAACCAA TTCTGAACAT TATCGCGAGC CCATTTATAC

4681 CTGAATATGG CTCATAACAC CCCTTGTTTG CCTGGCGGCA GTAGCGCGGT GGTCCCACCT

4741 GACCCCATGC CGAACTCAGA AGTGAAACGC CGTAGCGCCG ATGGTAGTGT GGGGACTCCC

4801 CATGCGAGAG TAGGGAACTG CCAGGCATCA AATAAAACGA AAGGCTCAGT CGAAAGACTG

4861 GGCCTTTCGC CCGGGCTAAT TATGGGGTGT CGCCCTTATT CGACTCTATA GTGAAGTTCC

4921 TATTCTCTAG AAAGTATAGG AACTTCTGAA GTGGGGATTT AAAGTCGGTG TCCGCGCTGA

4981 GGGTTTAATG GCGCGCCGCG GCCGCCCGCG GTGTTGGAAT AAAAATCAAC TATCATCTAC

5041 TAACTAGTAT TTACGTTACT AGTATATTAT CATATACGGT GTTAGAAGAT GACGCAAATG

5101 ATGAGAAATA GTCATCTAAA TTAGTGGAAG CTGAAACGCA AGGATTGATA ATGTAATAGG

5161 ATCAATGAAT ATTAACGCTA GCATTAAGTC CTCAGCGAGC ACGCTGCTTC ATGGAATGCG

5221 TGCGATGAGC GACCTCATGC TATACCTGAG AAAGCAACCT GACCTACAGG AAAGAGTTAC

5281 TCAAGAATAA GAATTTTCGT TTTAAAACCT AAGAGTCACT TTAAAATTTG TATACACTTA

5341 TTTTTTTTAT AACTTATTTA ATAATAAAAA TCATAAATCA TAAGAAATTC GCTTATTTAG

5401 AAGTGTCAAC AACGTATCTA CCAACGGAAT GCGTGCGAUG GAAGTACCTT CAAAGAATGG

5461 GGTCTTATCT TGTTTTGCAA GTACCACTGA GCAGGATAAT AATAGAAATG ATAATATACT

5521 ATAGTAGAGA TAACGTCGAT GACTTCCCAT ACTGTAATTG CTTTTAGTTG TGTATTTTTA

5581 GTGTGCAAGT TTCTGTAAAT CGATTAATTT TTTTTTCTTT CCTCTTTTTA TTAACCTTAA

5641 TTTTTATTTT AGATTCCTGA CTTCAACTCA AGACGCACAG ATATTATAAC ATCTGCATAA

5701 TAGGCATTTG CAAGAATTAC TCGTGAGTAA GGAAAGAGTG AGGAACTATC GCATACCTGC

5761 ATTTAAAGAT GCCGATTTGG GCGCGAATCC TTTATTTTGG CTTCACCCTC ATACTATTAT

5821 CAGGGCCAGA AAAAGGAAGT GTTTCCCTCC TTCTTGAATT GATGTTACCC TCATAAAGCA

5881 CGTGGCCTCT TATCGAGAAA GAAATTACCG TCGCTCGTGA TTTGTTTGCA AAAAGAACAA

5941 AACTGAAAAA ACCCAGACAC GCTCGACTTC CTGTCTTCCT ATTGATTGCA GCTTCCAATT

6001 TCGTCACACA ACAAGGTCCT AGCGACGGCT CACAGGTTTT GTAACAAGCA ATCGAAGGTT

6061 CTGGAATGGC GGGAAAGGGT TTAGTACCAC ATGCTATGAT GCCCACTGTG ATCTCCAGAG

6121 CAAAGTTCGT TCGATCGTAC TGTTACTCTC TCTCTTTCAA ACAGAATTGT CCGAATCGTG

6181 TGACAACAAC AGCCTGTTCT CACACACTCT TTTCTTCTAA CCAAGGGGGT GGTTTAGTTT

6241 AGTAGAACCT CGTGAAACTT ACATTTACAT ATATATAAAC TTGCATAAAT TGGTCAATGC

6301 AAGAAATACA TATTTGGTCT TTTCTAATTC GTAGTTTTTC AAGTTCTTAG ATGCTTTCTT

6361 TTTCTCTTTT TTACAGATCA TCAAGGAAGT AATTATCTAC TTTTTACAAC AAATATAAAA

6421 CAAATCTGTC AUAAAACAAT GATTAGCACC GAAAGCGATC TGCGTCGTCA GCTGGATGAA

6481 AATGTTCGTA GCGAAGCAGA TGAAAGCACC AAAGAAGAAT GTCCGTATAT TAACGCAGTT

6541 CAGAGCCATC ATCAGAACGT TCAAGAAATG AGCATTATTA TCAGCCTGGT GAAAAACATG

6601 AACGATATGA AAAGCATCAT CAGCATCTTT ACCGATCGCA ACATTAACAT CCTGCATATT

6661 GAAAGCCGTC TGGGTCGTCT GAATATGAAA AAACATACCG AAAAAAGCGA ATTTGAGCCG

6721 CTGGAACTGC TGGTTCATGT TGAAGTTCCG TGTATTGAAG TTGAACGTCT GCTGGAAGAA

6781 CTGAAAAGCT TTAGCAGCTA TCGTATTGTT CAGAATCCGC TGATGAATCT GCCGGAAGCA

6841 AAAAATCCGA CCCTGGATGA TAAAGTGCCG TGGTTTCCGC GTCATATTTC AGATCTGGAT

6901 AAAGTTAGCA ATAGCGTGCT GATGTATGGC AAAGAACTGG ATGCAGATCA TCCGGGTTTT

6961 AAAGATAAAG AATATCGCAA ACGTCGCATG ATGTTTGCAG ATATTGCACT GAACTATAAA

7021 TGGGGTCAGC AGATTCCGAT TGTGGAATAT ACCGAAATTG AAAAAACCAC CTGGGGTCGT

7081 ATTTATCGTG AACTGACCCG TCTGTATAAA ACCAGCGCAT GTCATGAATT TCAGAAAAAT

7141 CTGGGTCTGC TGCAGGATAA AGCAGGCTAT AATGAATTTG ATCTGCCGCA GCTGCAGGTT

7201 GTTAGCGATT TCCTGAAAGC ACGTACCGGT TTTTGTCTGC GTCCGGTTGC AGGTTATCTG

7261 AGCGCACGTG ATTTTCTGAG CGGTCTGGCA TTTCGTGTGT TTTATTGTAC CCAGTATATT

7321 CGTCATCAGG CCGATCCGTT TTATACTCCG GAACCGGATT GTTGTCATGA GCTGCTGGGC

7381 CATGTTCCGA TGCTGGCAGA TCCGAAATTT GCACGTTTTA GCCAAGAAAT TGGTCTGGCA

7441 AGCCTGGGCA CCAGTGATGA AGAAATCAAA AAACTGGCAA CCTGCTACTT CTTTACCATT

7501 GAATTTGGTC TGTGCCGTCA GGATAATCAG CTGAAAGCAT ATGGTGCAGG TCTGCTGAGC

7561 AGCGTTGCAG AACTGCAGCA TGCACTGAGC GATAAAGCCG TTATTAAACC GTTTATTCCG

7621 ATGAAAGTGA TCAACGAAGA ATGCCTGGTT ACCACCTTTC AGAATGGTTA TTTCGAAACC

7681 AGCAGCTTTG AAGATGCAAC CCGTCAGATG CGTGAATTTG TTCGTACCAT TAAACGTCCG

7741 TTTGATGTGC ATTATAATCC GTATACCCAG AGCATCGAAA TTATCAAAAC CCCGAAAAGC

7801 GTTGCCAAAC TGGTTCAGGA TCTGCAGTTT GAACTGACAG CAATTAATGA AAGCCTGCTG

7861 AAAATGAACA AAGAAATTCG TAGCCAGCAG TTTACCACCA ACAAAATTGT TACCGAAAAT

7921 CGCAGCAGCT AAATCGCGTG

SEQ ID NO: 13

pCfB2773

```
LOCUS       p2773_(pTY2-KlURA3-TAG-PGK1->HsTPH-NC(146-460))      7394 bp
DNA     circular
FEATURES             Location/Qualifiers
       terminator      6..176
                    /label=tCYC1; terminator of S. cerevisiae
```

```
     Misc._feature   224..283
                  /label=loxP
     terminator      complement(284..406)
                  /label=tURA3; terminator of K. lactis
     misc_structure  complement(407..457)
                  /label="Degradation tag"
     CDS             complement(458..1258)
                  /label=KlURA3 (minus STOP codon)
     promoter        complement(1259..1757)
                  /label=pURA3; promoter of K. lactis
     Misc._feature   1758..1811
                  /label=loxP
     Misc._feature   1951..2137
                  /label=TY2 3'
     rep_origin      complement(2924..3567)
                  /label=pUC ori
     CDS             complement(3670..4528)
                  /label=AmpR
     Misc._feature   4972..5200
                  /label=TY2 5'
     terminator      complement(5227..5421)
                  /label=tADH1; terminator of S. cerevisiae
     promoter        5440..6423
                  /label=pPGK1, promoter of S. cerevisiae
     CDS             6439..7386
                  /label=HsTPH2-NC(146-460)
ORIGIN
        1 CATTCATCCG CTCTAACCGA AAAGGAAGGA GTTAGACAAC CTGAAGTCTA GGTCCCTATT
       61 TATTTTTTTA TAGTTATGTT AGTATTAAGA ACGTTATTTA TATTTCAAAT TTTTCTTTTT
      121 TTTCTGTACA GACGCGTGTA CGCATGTAAC ATTATACTGA AAACCTTGCT TGAGAAATCG
      181 CGTCAGCTGA AGCTTCGTAC GCTGCAGGTC GACAACCCTT AATGTCGACA ACCCTTAATA
      241 TAACTTCGTA TAATGTATGC TATACGAAGT TATTAGGTCT AGAGATCCCA ATACAACAGA
      301 TCACGTGATC TTTTGTAAGA TGAAGTTGAA GTGAGTGTTG CACCGTGCCA ATGCAGGTGG
      361 CTATTAGATT AAATATGTGA TTTGTTCTAT TAAGTTTCCT GTATAATTAC AAATGAATAA
```

421 CGAAATGAGA CAAAGAAGAG AACCAATTTT TACAAGCATG GGGAGCGCTG ATTCTCTTTT
481 GGTACGCTTC CCATCCAGCA TTTCTGTATC TTTCACCTTC AACCTTAGGA TCTCTACCCT
541 TGGCGAAAAG TCCTCTGCCA ACAATGATGA TATCTGATCC ACCACTTACA ACTTCGTCGA
601 CGGTTCTGTA CTGCTGACCC AATGCATCGC CTTTGTCGTC TAAACCTACA CCTGGGGTCA
661 TGATTAGCCA ATCAAACCCT TCTTCTCTTC CTCCCATATC GTTCTGAGCA ATGAACCCAA
721 TAACGAAATC TTTATCACTC TTTGCAATAT CAACGGTACC CTTAGTATAT TCACCGTGTG
781 CTAGAGAACC CTTGGAAGAC AATTCAGCAA GCATCAATAA TCCCCTTGGT TCTTTGGTGA
841 CCTCTTGCGC ACCTTGTTTC AAGCCAGCAA CAATACCAGC ACCAGTAACC CCGTGGGCGT
901 TGGTGATATC AGACCATTCT GCGATACGGT AAACGCCCGA TGTATATTGT AATTTGACTG
961 TGTTACCGAT ATCGGCGAAT TTTCTGTCCT CAAATATCAA GAACTTGTAT TTCTCTGCCA
1021 ATGCTTTCAA TGGAACGACA GTACCCTCAT AACTGAAATC ATCCAAGATA TCAACGTGTG
1081 TTTTCAAAAG GCAAATGTAT GGACCCAACG TTTCAACAAG TTTCAATAGC TCATCAGTCG
1141 AACGAACGTC AAGAGAAGCA CACAAATTGG TCTTCTTTTC ATCCATTAAA CGTAAAAGTT
1201 TCGATGCAAC CGGACTTGCA TGAGTCTCAG CTCTACTGGT ATATGATTTT GTGGACATGG
1261 TGCAACTAAT TGACGGGAGT GTATTGACGC TGGCGTACTG GCTTTCACAA AATGGCCCAA
1321 TCACAACCAC ATCTTAGATA GTTGAAATGA CTTTAGATAA CATCAATTGA GATGAGCTTA
1381 ATCATGTCAA AGCTAAAAGT GTCACCATGA ACGACAATTC TTAAGCAAAT CACGTGATAT
1441 AGATCCACGA ATAACCACCA TTTGATGCTC GAGGCAAGTA ATGTGTGTAA AAAAATGCGT
1501 TACCACCATC CAATGCAGAC CGATCTTCTA CCCAGAATCA CATATATTTA TGTACCGAGT
1561 ACCTTTTTTC TATCTTCCAA TTGCTTCTCC CATATGATTG TCTCCGTAAG CTCGAAATTT
1621 CTAAGTTGGA TTTTAATCTT CACGCAGGAT GACAGTTCGA TGAGCTTCTG AGGAGTGTTT
1681 AGAACATAAT CAGTTTATCC ATGGTCTATC TCTTCTTGTC GCTTTTTCTC CTCGATAGAA
1741 CCTAAATAAA ACGAGCTCTC GAGAACCCTT AATATAACTT CGTATAATGT ATGCTATACG
1801 AAGTTATTAG GTAGGTGATA TCAGATCCAC TAGTGGCCTA TGCACCCAAT TCGCCCTATA
1861 GTGAGTCGTA TTACGCGCGC TCACTGGCCG TCGTTTTACA ACGTCGTGAC TGGGAAAACC
1921 CTGGCGTTAC CCCTGCAGGA CAGACGTCAT TAGTGCTGAG GCATTAATTG ATCAATATAA
1981 AATGATGATA ATAATATTTA TAGAATTGTG TAGAATTGCA GATTCCCTTT TATGGATTCC
2041 TAAATCCTGA GGAGAACTTC TAGTATATTC TACATACCTA ATATTATTGC CTTATTAAAA
2101 ATGGAATCCC AACAATTACA TCAAAATCCA CATTCTCAGA TCTGCGGCCG CACTCAGACC
2161 TGAAGTGAAG TTCCTATACT TTCTAGAGAA TAGGAACTTC TATAGTGAGT CGAATAAGGG
2221 CGACACAAAA TTTATTCTAA ATGCATAATA AATACTGATA ACATCTTATA GTTTGTATTA
2281 TATTTTGTAT TATCGTTGAC ATGTATAATT TTGATATCAA AAACTGATTT TCCCTTTATT
2341 ATTTTCGAGA TTTATTTTCT TAATTCTCTT TAACAAACTA GAAATATTGT ATATACAAAA
2401 AATCATAAAT AATAGATGAA TAGTTTAATT ATAGGTGTTC ATCAATCGAA AAAGCAACGT

2461 ATCTTATTTA AAGTGCGTTG CTTTTTTCTC ATTTATAAGG TTAAATAATT CTCATATATC

2521 AAGCAAAGTG ACAGGCGCCC TTAAATATTC TGACAAATGC TCTTTCCCTA AACTCCCCCC

2581 ATAAAAAAAC CCGCCGAAGC GGGTTTTTAC GTTATTTGCG GATTAACGAT TACTCGTTAT

2641 CAGAACCGCC CAGGGGGCCC GAGCTTAAGA CTGGCCGTCG TTTTACAACA CAGAAAGAGT

2701 TTGTAGAAAC GCAAAAAGGC CATCCGTCAG GGGCCTTCTG CTTAGTTTGA TGCCTGGCAG

2761 TTCCCTACTC TCGCCTTCCG CTTCCTCGCT CACTGACTCG CTGCGCTCGG TCGTTCGGCT

2821 GCGGCGAGCG GTATCAGCTC ACTCAAAGGC GGTAATACGG TTATCCACAG AATCAGGGGA

2881 TAACGCAGGA AAGAACATGT GAGCAAAAGG CCAGCAAAAG GCCAGGAACC GTAAAAAGGC

2941 CGCGTTGCTG GCGTTTTTCC ATAGGCTCCG CCCCCCTGAC GAGCATCACA AAAATCGACG

3001 CTCAAGTCAG AGGTGGCGAA ACCCGACAGG ACTATAAAGA TACCAGGCGT TTCCCCCTGG

3061 AAGCTCCCTC GTGCGCTCTC CTGTTCCGAC CCTGCCGCTT ACCGGATACC TGTCCGCCTT

3121 TCTCCCTTCG GGAAGCGTGG CGCTTTCTCA TAGCTCACGC TGTAGGTATC TCAGTTCGGT

3181 GTAGGTCGTT CGCTCCAAGC TGGGCTGTGT GCACGAACCC CCCGTTCAGC CCGACCGCTG

3241 CGCCTTATCC GGTAACTATC GTCTTGAGTC CAACCCGGTA AGACACGACT TATCGCCACT

3301 GGCAGCAGCC ACTGGTAACA GGATTAGCAG AGCGAGGTAT GTAGGCGGTG CTACAGAGTT

3361 CTTGAAGTGG TGGGCTAACT ACGGCTACAC TAGAAGAACA GTATTTGGTA TCTGCGCTCT

3421 GCTGAAGCCA GTTACCTTCG GAAAAAGAGT TGGTAGCTCT TGATCCGGCA AACAAACCAC

3481 CGCTGGTAGC GGTGGTTTTT TTGTTTGCAA GCAGCAGATT ACGCGCAGAA AAAAAGGATC

3541 TCAAGAAGAT CCTTTGATCT TTTCTACGGG GTCTGACGCT CAGTGGAACG ACGCGCGCGT

3601 AACTCACGTT AAGGGATTTT GGTCATGAGC TTGCGCCGTC CCGTCAAGTC AGCGTAATGC

3661 TCTGCTTTTA CCAATGCTTA ATCAGTGAGG CACCTATCTC AGCGATCTGT CTATTTCGTT

3721 CATCCATAGT TGCCTGACTC CCCGTCGTGT AGATAACTAC GATACGGGAG GGCTTACCAT

3781 CTGGCCCCAG CGCTGCGATG ATACCGCGAG AACCACGCTC ACCGGCTCCG GATTTATCAG

3841 CAATAAACCA GCCAGCCGGA AGGGCCGAGC GCAGAAGTGG TCCTGCAACT TTATCCGCCT

3901 CCATCCAGTC TATTAATTGT TGCCGGGAAG CTAGAGTAAG TAGTTCGCCA GTTAATAGTT

3961 TGCGCAACGT TGTTGCCATC GCTACAGGCA TCGTGGTGTC ACGCTCGTCG TTTGGTATGG

4021 CTTCATTCAG CTCCGGTTCC CAACGATCAA GGCGAGTTAC ATGATCCCCC ATGTTGTGCA

4081 AAAAAGCGGT TAGCTCCTTC GGTCCTCCGA TCGTTGTCAG AAGTAAGTTG GCCGCAGTGT

4141 TATCACTCAT GGTTATGGCA GCACTGCATA ATTCTCTTAC TGTCATGCCA TCCGTAAGAT

4201 GCTTTTCTGT GACTGGTGAG TACTCAACCA AGTCATTCTG AGAATAGTGT ATGCGGCGAC

4261 CGAGTTGCTC TTGCCCGGCG TCAATACGGG ATAATACCGC GCCACATAGC AGAACTTTAA

4321 AAGTGCTCAT CATTGGAAAA CGTTCTTCGG GGCGAAAACT CTCAAGGATC TTACCGCTGT

4381 TGAGATCCAG TTCGATGTAA CCCACTCGTG CACCCAACTG ATCTTCAGCA TCTTTTACTT

4441 TCACCAGCGT TTCTGGGTGA GCAAAAACAG GAAGGCAAAA TGCCGCAAAA AAGGGAATAA

4501 GGGCGACACG GAAATGTTGA ATACTCATAT TCTTCCTTTT TCAATATTAT TGAAGCATTT

4561 ATCAGGGTTA TTGTCTCATG AGCGGATACA TATTTGAATG TATTTAGAAA AATAAACAAA

4621 TAGGGGTCAG TGTTACAACC AATTAACCAA TTCTGAACAT TATCGCGAGC CCATTTATAC

4681 CTGAATATGG CTCATAACAC CCCTTGTTTG CCTGGCGGCA GTAGCGCGGT GGTCCCACCT

4741 GACCCCATGC CGAACTCAGA AGTGAAACGC CGTAGCGCCG ATGGTAGTGT GGGGACTCCC

4801 CATGCGAGAG TAGGGAACTG CCAGGCATCA AATAAAACGA AAGGCTCAGT CGAAAGACTG

4861 GGCCTTTCGC CCGGGCTAAT TATGGGGTGT CGCCCTTATT CGACTCTATA GTGAAGTTCC

4921 TATTCTCTAG AAAGTATAGG AACTTCTGAA GTGGGGATTT AAAGTCGGTG TCCGCGCTGA

4981 GGGTTTAATG GCGCGCCGCG GCCGCCCGCG GTGTTGGAAT AAAAATCAAC TATCATCTAC

5041 TAACTAGTAT TTACGTTACT AGTATATTAT CATATACGGT GTTAGAAGAT GACGCAAATG

5101 ATGAGAAATA GTCATCTAAA TTAGTGGAAG CTGAAACGCA AGGATTGATA ATGTAATAGG

5161 ATCAATGAAT ATTAACGCTA GCATTAAGTC CTCAGCGAGC ACGCTGCTTC ATGGAATGCG

5221 TGCGATGAGC GACCTCATGC TATACCTGAG AAAGCAACCT GACCTACAGG AAAGAGTTAC

5281 TCAAGAATAA GAATTTTCGT TTTAAAACCT AAGAGTCACT TTAAAATTTG TATACACTTA

5341 TTTTTTTTAT AACTTATTTA ATAATAAAAA TCATAAATCA TAAGAAATTC GCTTATTTAG

5401 AAGTGTCAAC AACGTATCTA CCAACGGAAT GCGTGCGAUG GAAGTACCTT CAAAGAATGG

5461 GGTCTTATCT TGTTTTGCAA GTACCACTGA GCAGGATAAT AATAGAAATG ATAATATACT

5521 ATAGTAGAGA TAACGTCGAT GACTTCCCAT ACTGTAATTG CTTTTAGTTG TGTATTTTTA

5581 GTGTGCAAGT TTCTGTAAAT CGATTAATTT TTTTTTCTTT CCTCTTTTTA TTAACCTTAA

5641 TTTTTATTTT AGATTCCTGA CTTCAACTCA AGACGCACAG ATATTATAAC ATCTGCATAA

5701 TAGGCATTTG CAAGAATTAC TCGTGAGTAA GGAAAGAGTG AGGAACTATC GCATACCTGC

5761 ATTTAAAGAT GCCGATTTGG GCGCGAATCC TTTATTTTGG CTTCACCCTC ATACTATTAT

5821 CAGGGCCAGA AAAAGGAAGT GTTTCCCTCC TTCTTGAATT GATGTTACCC TCATAAAGCA

5881 CGTGGCCTCT TATCGAGAAA GAAATTACCG TCGCTCGTGA TTTGTTTGCA AAAAGAACAA

5941 AACTGAAAAA ACCCAGACAC GCTCGACTTC CTGTCTTCCT ATTGATTGCA GCTTCCAATT

6001 TCGTCACACA ACAAGGTCCT AGCGACGGCT CACAGGTTTT GTAACAAGCA ATCGAAGGTT

6061 CTGGAATGGC GGGAAAGGGT TTAGTACCAC ATGCTATGAT GCCCACTGTG ATCTCCAGAG

6121 CAAAGTTCGT TCGATCGTAC TGTTACTCTC TCTCTTTCAA ACAGAATTGT CCGAATCGTG

6181 TGACAACAAC AGCCTGTTCT CACACACTCT TTTCTTCTAA CCAAGGGGGT GGTTTAGTTT

6241 AGTAGAACCT CGTGAAACTT ACATTTACAT ATATATAAAC TTGCATAAAT TGGTCAATGC

6301 AAGAAATACA TATTTGGTCT TTTCTAATTC GTAGTTTTTC AAGTTCTTAG ATGCTTTCTT

6361 TTTCTCTTTT TTACAGATCA TCAAGGAAGT AATTATCTAC TTTTTACAAC AAATATAAAA

6421 CAAATCTGTC AUAAAACAAT GGAACTGGAA GATGTTCCGT GGTTTCCGCG TAAAATTAGC

6481 GAACTGGATA AATGTAGCCA TCGTGTTCTG ATGTATGGTA GTGAACTGGA TGCAGATCAT

```
6541 CCGGGTTTTA AAGATAATGT TTATCGTCAG CGTCGCAAAT ATTTCGTTGA TGTTGCAATG
6601 GGTTACAAAT ATGGTCAGCC GATTCCGCGT GTTGAATATA CCGAAGAAGA AACCAAAACC
6661 TGGGGTGTTG TTTTTCGTGA ACTGAGCAAA CTGTATCCGA CCCATGCATG TCGTGAATAT
6721 CTGAAAAACT TTCCGCTGCT GACCAAATAT TGTGGTTATC GTGAAGATAA CGTTCCGCAG
6781 CTGGAAGATG TGAGCATGTT TCTGAAAGAA CGTAGCGGTT TTACCGTTCG TCCGGTTGCA
6841 GGTTATCTGA GTCCGCGTGA TTTTCTGGCA GGTCTGGCAT ATCGTGTTTT TCATTGTACC
6901 CAGTATATTC GCCATGGTAG CGATCCGCTG TATACTCCGG AACCGGATAC CTGTCATGAA
6961 CTGCTGGGTC ATGTGCCGCT GCTGGCAGAT CCGAAATTTG CACAGTTTAG CCAAGAAATT
7021 GGTCTGGCAA GCCTGGGTGC AAGTGATGAA GATGTGCAGA AACTGGCAAC CTGTTATTTC
7081 TTTACCATTG AATTTGGCCT GTGCAAACAA GAGGGTCAGC TGCGTGCATA TGGTGCAGGT
7141 CTGCTGAGCA GCATTGGTGA ACTGAAACAT GCACTGAGCG ATAAAGCATG TGTTAAAGCA
7201 TTTGATCCGA AAACCACCTG TCTGCAAGAA TGTCTGATTA CCACCTTTCA AGAAGCCTAT
7261 TTCGTTAGCG AAAGCTTTGA AGAGGCCAAA GAAAAAATGC GCGATTTTGC CAAAAGCATT
7321 ACCCGTCCGT TTAGCGTTTA TTTCAATCCG TATACCCAGA GCATCGAGAT CCTGAAAGAT
7381 ACCTAAATCG CGTG
```

SEQ ID NO: 14

pCfB3337

```
LOCUS       p3337 (pESC-NatMXsyn-HsASMT<-PTEF1) 6547 bp     DNA     circular
FEATURES             Location/Qualifiers
       misc_feature    join(4841..4845,1..5)
                     /label=USER cloning site
       rep_origin      189..856
                     /label=pUC ori
       CDS             complement(1007..1864)
                     /label=AmpR
       rep_origin      1998..3153
                     /label=2 micron ori
       misc_feature    3533..3919
                    /label=pTEF1; promoter of A. gossypii
       CDS             3921..4493
                    /label=nat; Nourseothricin acetyltransferase
       terminator     4494..4742
                    /label=tTEF1; terminator of A. gossypii
```

```
     terminator      complement(4854..5048)
                     /label=tADH1; terminator of S. cerevisiae
     CDS             complement(5067..6104)
                     /label=HsASMT
     promoter        complement(6120..6539)
                     /label=pTEF1; promoter of S. cerevisiae
ORIGIN
        1 CATTCCAGCT GCATTAATGA ATCGGCCAAC GCGCGGGGAG AGGCGGTTTG CGTATTGGGC
       61 GCTCTTCCGC TTCCTCGCTC ACTGACTCGC TGCGCTCGGT CGTTCGGCTG CGGCGAGCGG
      121 TATCAGCTCA CTCAAAGGCG GTAATACGGT TATCCACAGA ATCAGGGGAT AACGCAGGAA
      181 AGAACATGTG AGCAAAAGGC CAGCAAAAGG CCAGGAACCG TAAAAAGGCC GCGTTGCTGG
      241 CGTTTTTCCA TAGGCTCCGC CCCCCTGACG AGCATCACAA AAATCGACGC TCAAGTCAGA
      301 GGTGGCGAAA CCCGACAGGA CTATAAAGAT ACCAGGCGTT TCCCCCTGGA AGCTCCCTCG
      361 TGCGCTCTCC TGTTCCGACC CTGCCGCTTA CCGGATACCT GTCCGCCTTT CTCCCTTCGG
      421 GAAGCGTGGC GCTTTCTCAT AGCTCACGCT GTAGGTATCT CAGTTCGGTG TAGGTCGTTC
      481 GCTCCAAGCT GGGCTGTGTG CACGAACCCC CCGTTCAGCC CGACCGCTGC GCCTTATCCG
      541 GTAACTATCG TCTTGAGTCC AACCCGGTAA GACACGACTT ATCGCCACTG GCAGCAGCCA
      601 CTGGTAACAG GATTAGCAGA GCGAGGTATG TAGGCGGTGC TACAGAGTTC TTGAAGTGGT
      661 GGCCTAACTA CGGCTACACT AGAAGGACAG TATTTGGTAT CTGCGCTCTG CTGAAGCCAG
      721 TTACCTTCGG AAAAAGAGTT GGTAGCTCTT GATCCGGCAA ACAAACCACC GCTGGTAGCG
      781 GTGGTTTTTT TGTTTGCAAG CAGCAGATTA CGCGCAGAAA AAAAGGATCT CAAGAAGATC
      841 CTTTGATCTT TTCTACGGGG TCTGACGCTC AGTGGAACGA AAACTCACGT TAAGGGATTT
      901 TGGTCATGAG ATTATCAAAA AGGATCTTCA CCTAGATCCT TTTAAATTAA AAATGAAGTT
      961 TTAAATCAAT CTAAAGTATA TATGAGTAAA CTTGGTCTGA CAGTTACCAA TGCTTAATCA
     1021 GTGAGGCACC TATCTCAGCG ATCTGTCTAT TTCGTTCATC CATAGTTGCC TGACTCCCCG
     1081 TCGTGTAGAT AACTACGATA CGGGAGGGCT TACCATCTGG CCCCAGTGCT GCAATGATAC
     1141 CGCGAGACCC ACGCTCACCG GCTCCAGATT TATCAGCAAT AAACCAGCCA GCCGGAAGGG
     1201 CCGAGCGCAG AAGTGGTCCT GCAACTTTAT CCGCCTCCAT CCAGTCTATT AATTGTTGCC
     1261 GGGAAGCTAG AGTAAGTAGT TCGCCAGTTA ATAGTTTGCG CAACGTTGTT GCCATTGCTA
     1321 CAGGCATCGT GGTGTCACGC TCGTCGTTTG GTATGGCTTC ATTCAGCTCC GGTTCCCAAC
     1381 GATCAAGGCG AGTTACATGA TCCCCCATGT TGTGCAAAAA AGCGGTTAGC TCCTTCGGTC
     1441 CTCCGATCGT TGTCAGAAGT AAGTTGGCCG CAGTGTTATC ACTCATGGTT ATGGCAGCAC
     1501 TGCATAATTC TCTTACTGTC ATGCCATCCG TAAGATGCTT TTCTGTGACT GGTGAGTACT
     1561 CAACCAAGTC ATTCTGAGAA TAGTGTATGC GGCGACCGAG TTGCTCTTGC CCGGCGTCAA
```

1621 TACGGGATAA TACCGCGCCA CATAGCAGAA CTTTAAAAGT GCTCATCATT GGAAAACGTT

1681 CTTCGGGGCG AAAACTCTCA AGGATCTTAC CGCTGTTGAG ATCCAGTTCG ATGTAACCCA

1741 CTCGTGCACC CAACTGATCT TCAGCATCTT TTACTTTCAC CAGCGTTTCT GGGTGAGCAA

1801 AAACAGGAAG GCAAAATGCC GCAAAAAAGG GAATAAGGGC GACACGGAAA TGTTGAATAC

1861 TCATACTCTT CCTTTTTCAA TATTATTGAA GCATTTATCA GGGTTATTGT CTCATGAGCG

1921 GATACATATT TGAATGTATT TAGAAAAATA AACAAATAGG GGTTCCGCGC ACATTTCCCC

1981 GAAAAGTGCC ACCTGAACGA AGCATCTGTG CTTCATTTTG TAGAACAAAA ATGCAACGCG

2041 AGAGCGCTAA TTTTTCAAAC AAAGAATCTG AGCTGCATTT TTACAGAACA GAAATGCAAC

2101 GCGAAAGCGC TATTTTACCA ACGAAGAATC TGTGCTTCAT TTTTGTAAAA CAAAAATGCA

2161 ACGCGAGAGC GCTAATTTTT CAAACAAAGA ATCTGAGCTG CATTTTTACA GAACAGAAAT

2221 GCAACGCGAG AGCGCTATTT TACCAACAAA GAATCTATAC TTCTTTTTTG TTCTACAAAA

2281 ATGCATCCCG AGAGCGCTAT TTTTCTAACA AAGCATCTTA GATTACTTTT TTTCTCCTTT

2341 GTGCGCTCTA TAATGCAGTC TCTTGATAAC TTTTTGCACT GTAGGTCCGT TAAGGTTAGA

2401 AGAAGGCTAC TTTGGTGTCT ATTTTCTCTT CCATAAAAAA AGCCTGACTC CACTTCCCGC

2461 GTTTACTGAT TACTAGCGAA GCTGCGGGTG CATTTTTTCA AGATAAAGGC ATCCCCGATT

2521 ATATTCTATA CCGATGTGGA TTGCGCATAC TTTGTGAACA GAAAGTGATA GCGTTGATGA

2581 TTCTTCATTG GTCAGAAAAT TATGAACGGT TTCTTCTATT TTGTCTCTAT ATACTACGTA

2641 TAGGAAATGT TTACATTTTC GTATTGTTTT CGATTCACTC TATGAATAGT TCTTACTACA

2701 ATTTTTTTGT CTAAAGAGTA ATACTAGAGA TAAACATAAA AAATGTAGAG GTCGAGTTTA

2761 GATGCAAGTT CAAGGAGCGA AAGGTGGATG GGTAGGTTAT ATAGGGATAT AGCACAGAGA

2821 TATATAGCAA AGAGATACTT TTGAGCAATG TTTGTGGAAG CGGTATTCGC AATATTTTAG

2881 TAGCTCGTTA CAGTCCGGTG CGTTTTTGGT TTTTTGAAAG TGCGTCTTCA GAGCGCTTTT

2941 GGTTTTCAAA AGCGCTCTGA AGTTCCTATA CTTTCTAGAG AATAGGAACT TCGGAATAGG

3001 AACTTCAAAG CGTTTCCGAA AACGAGCGCT TCCGAAAATG CAACGCGAGC TGCGCACATA

3061 CAGCTCACTG TTCACGTCGC ACCTATATCT GCGTGTTGCC TGTATATATA TATACATGAG

3121 AAGAACGGCA TAGTGCGTGT TTATGCTTAA ATGCGTACTT ATATGCGTCT ATTTATGTAG

3181 GATGAAAGGT AGTCTAGTAC CTCCTGTGAT ATTATCCCAT TCCATGCGGG GTATCGTATG

3241 CTTCCTTCAG CACTACCCTT TAGCTGTTCT ATATGCTGCC ACTCCTCAAT TGGATTAGTC

3301 TCATCCTTCA ATGCTATCAT TTCCTTTGAT ATTGGATCAT ACTAAGAAAC CATTATTATC

3361 ATGACATTAA CCTATAAAAA TAGGCGTATC ACGAGGCCCT TTCGTCTCGC GCGTTTCGGT

3421 GATGACGGTG AAAACCTCTG ACACATGCAG CTCCCGGAGA CGGTCACAGC TTGTCTGTAA

3481 GCGGATGCCG GGAGCAGACA AGCCCGTCAG GGCGCGTCAG CGGGATTTAA ATGATCTGTT

3541 TAGCTTGCCT CGTCCCCGCC GGGTCACCCG GCCAGCGACA TGGAGGCCCA GAATACCCTC

3601 CTTGACAGTC TTGACGTGCG CAGCTCAGGG GCATGATGTG ACTGTCGCCC GTACATTTAG

3661 CCCATACATC CCCATGTATA ATCATTTGCA TCCATACATT TTGATGGCCG CACGGCGCGA

3721 AGCAAAAATT ACGGCTCCTC GCTGCAGACC TGCGAGCAGG GAAACGCTCC CCTCACAGAC

3781 GCGTTGAATT GTCCCCACGC CGCGCCCCTG TAGAGAAATA TAAAAGGTTA GGATTTGCCA

3841 CTGAGGTTCT TCTTTCATAT ACTTCCTTTT AAAATCTTGC TAGGATACAG TTCTCACATC

3901 ACATCCGAAC ATAAACAACC ATGGGTACCA CTCTTGACGA CACGGCTTAC CGGTACCGCA

3961 CCAGTGTCCC GGGGGACGCC GAGGCCATCG AGGCACTGGA TGGGTCCTTC ACCACCGACA

4021 CCGTTTTCCG CGTCACCGCC ACCGGGGACG GCTTCACCCT GCGGGAGGTG CCGGTGGACC

4081 CGCCCCTGAC CAAGGTGTTC CCCGACGACG AATCGGACGA CGAATCGGAC GACGGGGAGG

4141 ACGGCGACCC GGACTCCCGG ACGTTCGTCG CGTACGGGGA CGACGGCGAC CTGGCGGGCT

4201 TCGTGGTCGT CTCGTACTCC GGCTGGAACC GCCGGCTGAC CGTCGAGGAC ATCGAGGTCG

4261 CCCCGGAGCA CCGGGGGCAC GGGGTCGGGC GCGCGTTGAT GGGGCTCGCG ACGGAGTTCG

4321 CCCGCGAGCG GGGCGCCGGG CACCTCTGGC TGGAGGTCAC CAACGTCAAC GCACCGGCGA

4381 TCCACGCGTA CCGGCGGATG GGGTTCACCC TCTGCGGCCT GGACACCGCC CTGTACGACG

4441 GCACCGCCTC GGACGGCGAG CAGGCGCTCT ACATGAGCAT GCCCTGCCCC TAATCAGTAC

4501 TGACAATAAA AAGATTCTTG TTTTCAAGAA CTTGTCATTT GTATAGTTTT TTTATATTGT

4561 AGTTGTTCTA TTTTAATCAA ATGTTAGCGT GATTTATATT TTTTTTCGCC TCGACATCAT

4621 CTGCCCAGAT GCGAAGTTAA GTGCGCAGAA AGTAATATCA TGCGTCAATC GTATGTGAAT

4681 GCTGGTCGCT ATACTGCTGT CGATTCGATA CTAACGCCGC CATCCAGTGT CGAAAACGAG

4741 CTGCGGCCGC CTACAGGGCG CGTCGCGCCA TTCGCCATTC AGGCTGCGCA ACTGTTGGGA

4801 AGGGCGATCG GTGCGGGCCT CTTCGCTATT ACGCCAGCTG GAATGCGTGC GATGAGCGAC

4861 CTCATGCTAT ACCTGAGAAA GCAACCTGAC CTACAGGAAA GAGTTACTCA AGAATAAGAA

4921 TTTTCGTTTT AAAACCTAAG AGTCACTTTA AAATTTGTAT ACACTTATTT TTTTTATAAC

4981 TTATTTAATA ATAAAAATCA TAAATCATAA GAAATTCGCT TATTTAGAAG TGTCAACAAC

5041 GTATCTACCA ACGGAATGCG TGCGATTTAT TTACGTGCCA GGATTGCATC ATAAATGGCA

5101 CCGGTTTTTT TGAACTGAAA ATCACGAAAA CCTGCACTGC TCAGCAGCAT ATGATAATGG

5161 GTCGGGGTAC GTTCTTGACC TTCGGTCTGA ACCAGCATAT TCAGGCTATA CAGCTGGGTC

5221 AGCAGCGGAC CACGACGATC TTCATCCAGC AGGCTTTCAA TAACCAGAAT GCCACCACCC

5281 GGTTTACAGG TATGATAAAT ACGTTCCAGC AGATGGCTGC ATTTACCATC TGCCCAATCA

5341 TGCAGAACAC GTGCCAGAAT ATACAGATCT GCTTCCGGCA GCGGATCTTT GAAAAAATCG

5401 CCCTCTTGAA AGTCGATTTG TTCCTCTTCT TGAAAGCTAA AATGCTGTTT TGCGGTCCAC

5461 ACAACTTCCG GAATATCAAA AACGGTAATT TTACAACCCG GATACAGGCT CATACATTCT

5521 TTTGCCAGTG CACCGGCACC ACCACCCAGA TCACACATCA GCGGAAAAAC GCTCAGATCA

5581 AATGCGGTCA GAACGCTACG ACCATTAACG CTCCAAACCT CTTGCAGTGC CTGCATAAAC

5641 TGCAGACGTT CACCTTCGCT ACGATAAATT GCGGTAAACA GTTCTTCTGC CGGAACACCA

5701 AAGGTTTCCA GATACTGATT ACGACCTTCA CGAACTGCAT CTGCCAGATG ACCCCAACAA
5761 CGATAGCTGG TACGACCCAT ATATTTCAGC ATGCTACACT GGCTGGTCGG GCTAACGGTG
5821 GTCAGATAAT CGCTGCTCAG TTCGGTATTA CGATAAAATG CTTTACCACC ACGGGTTTCA
5881 ACTTTCAGCA GTTTCAGGCT AACACAAATA TCCAGCAGCA GTTCGGTGCC ATGTGCGCTT
5941 GCACGAACAC CGGCTGCAAC TGCTGCAACA TCCAGCGGAC CCGGTGCTTC TGCCAGCAGA
6001 TCAAAAACAC CCAGTTCACA TGCTGCAAAC AGAACCTGGC TAACCATAAA ACCATTGGCA
6061 TAATCATTCA GCAGACGATA TGCCTGATCT TCGCTGCTAC CCATTGTTTT ACCTGCACTT
6121 TGTAATTAAA ACTTAGATTA GATTGCTATG CTTTCTTTCT AATGAGCAAG AAGTAAAAAA
6181 AGTTGTAATA GAACAAGAAA AATGAAACTG AAACTTGAGA AATTGAAGAC CGTTTATTAA
6241 CTTAAATATC AATGGGAGGT CATCGAAAGA GAAAAAAATC AAAAAAAAAA ATTTTCAAGA
6301 AAAAGAAACG TGATAAAAAT TTTTATTGCC TTTTTCGACG AAGAAAAAGA AACGAGGCGG
6361 TCTCTTTTTT CTTTTCCAAA CCTTTAGTAC GGGTAATTAA CGACACCCTA GAGGAAGAAA
6421 GAGGGGAAAT TTAGTATGCT GTGCTTGGGT GTTTTGAAGT GGTACGGCGA TGCGCGGAGT
6481 CCGAGAAAAT CTGGAAGAGT AAAAAAGGAG TAGAAACATT TTGAAGCTAT GGTGTGTGCA
6541 TCGCGTG

Supplementary References

[1] Nørholm, M.H.H., A mutant Pfu DNA polymerase designed for advanced uracil-excision DNA engineering. *BMC Biotechnol.* 2010, *10*, 21.

[2] Jensen, N.B., Strucko, T., Kildegaard, K.R., David, F., et al., EasyClone: method for iterative chromosomal integration of multiple genes in Saccharomyces cerevisiae. *FEMS Yeast Res.* 2014, *14*, 238–48.

[3] Gietz, R.D., Schiestl, R.H., High-efficiency yeast transformation using the LiAc/SS carrier DNA/PEG method. *Nat. Protoc.* 2007, *2*, 31–4.

[4] Hoffman, C.S., Winston, F., A ten-minute DNA preparation from yeast efficiently releases autonomous plasmids for transformation of Escherichia coli. *Gene* 1987, *57*, 267–72.

[5] Partow, S., Siewers, V., Bjørn, S., Nielsen, J., et al., Characterization of different promoters for designing a new expression vector in Saccharomyces cerevisiae. *Yeast* 2010, 955–964.

[6] Chen, Y., Daviet, L., Schalk, M., Siewers, V., et al., Establishing a platform cell factory through engineering of yeast acetyl-CoA metabolism. *Metab. Eng.* 2013, *15*, 48–54.

[7] Knight, E.M., Zhu, J., Forster, J., Luo, H., Microorganisms for the production of melatonin. 2013.
